# Single‐cell transcriptomics reveal the intratumoral landscape of infiltrated T‐cell subpopulations in oral squamous cell carcinoma

**DOI:** 10.1002/1878-0261.12910

**Published:** 2021-03-09

**Authors:** Jingtao Chen, Jiefeng Yang, Huan Li, Zhongyuan Yang, Xing Zhang, Xiyuan Li, Jia Wang, Ying Zhang, Shuwei Chen, Ming Song

**Affiliations:** ^1^ Department of Head and Neck Surgery Sun Yat‐sen University Cancer Center Guangzhou China; ^2^ State Key Laboratory of Oncology in South China Guangzhou China; ^3^ Collaborative Innovation Center for Cancer Medicine Guangzhou China; ^4^ Department of Experimental Research Sun Yat‐sen University Cancer Center Guangzhou China; ^5^ Department of Intensive Care Unit Sun Yat‐sen University Cancer Center Guangzhou China

**Keywords:** oral squamous cell carcinoma, single‐cell sequencing, tumor‐infiltrating lymphocytes, T‐cell exhaustion, cancer immunology

## Abstract

Systematic analysis of tumor‐infiltrating lymphocytes is essential for the development of new cancer treatments and the prediction of clinical responses to immunotherapy. Immunomodulatory drugs are used for the treatment of oral squamous cell carcinoma (OSCC), depending on immune infiltration profiles of the tumor microenvironment. In this study, we isolated 11,866 single T cells from tumors and paired adjacent normal tissues of three patients with OSCC. Using single‐cell RNA sequencing, we identified 14 distinct T‐cell subpopulations within the tumors and 5 T‐cell subpopulations in the adjacent normal tissues and delineated their developmental trajectories. Exhausted CD8^+^ T cells and regulatory CD4^+^ T cells (CD4^+^ Tregs) were enriched in OSCC tumors, potentially linked to tumor immunosuppression. Programmed death protein 1 (PD‐1) and cytotoxic T lymphocyte‐associated protein 4 (CTLA4) were identified as marker genes in exhausted CD8^+^ T cells, whereas forkhead box P3 (FOXP3) and CTLA4 were identified as markers of CD4^+^ Tregs. Furthermore, our data revealed that thymocyte selection‐associated high‐mobility group box (TOX) may be a key regulator of T‐cell dysfunction in the OSCC microenvironment. Overexpression of TOX upregulated expression of genes related to T‐cell dysfunction. *In vitro* experiments demonstrated that cytotoxic activity and proliferation efficiency of CD8^+^ T cells overexpressing PD‐1 or TOX were reduced. Notable, the transcription factor PRDM1 was found to transactivate TOX expression via a binding motif in the *TOX* promoter. Our findings provide valuable insight into the functional states and heterogeneity of T‐cell populations in OSCC that could advance the development of novel therapeutic strategies.

## Graphical abstract

Using single‐cell RNA sequencing, we identified 14 distinct T‐cell subpopulations from 11,866 T cells in OSCC. Our data revealed that exhausted CD8^+^ T cells and CD4^+^ Tregs were enriched in OSCC and TOX may be a key regulator of T‐cell dysfunction. Notable, the transcription factor PRDM1 was found to transactivate TOX expression via a binding motif in the TOX promoter.

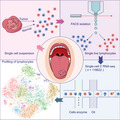

**Abbreviations**
BSA: bovine serum albumin
CFSE: carboxyfluorescein succinimidyl amino ester
FACS: fluorescence‐activated cell sorting
GO: gene ontology analysis
HPV: human papillomavirus
IC: immune checkpoint
IFN‐γ: interferon‐gamma
IHC: immunohistochemistry
OE: overexpressing
OSCC: oral squamous cell carcinoma
PD‐1: programmed death protein 1
PD‐L1: programmed death protein ligand 1
PRDM1: positive regulatory domain 1
scRNA‐seq: single‐cell RNA sequencing
T_EM_: effector memory T cell
T_EX_: exhausted T cell
TME: tumor microenvironment
TOX: thymocyte selection‐associated high‐mobility group box
Treg: regulatory T cell
T_RM_: tissue‐resident memory T cell
t‐SNE: T‐distributed stochastic neighbor embedding

## 1 Introduction

Genomic and transcriptomic studies have revealed the disease subtypes, aberrant regulatory programs, and driving mutations for many major human tumors [1, 2]. However, these studies relied on profiling technologies that analyzed tumors in bulk with limited ability to capture intratumoral heterogeneity. Substantial evidence indicates that immune infiltration of the tumor microenvironment (TME) and intratumoral heterogeneity and interactions among malignant and nonmalignant cells within the TME are critical to diverse aspects of tumor biology [2, 3].

With advances in microfluidics, the use of single‐cell RNA sequencing (scRNA‐seq) has enabled simultaneous profiling of thousands of cells from a biopsy sample at the single‐cell level. Single‐cell transcriptome analysis applied to cancerous and immune cells from melanoma patients revealed a typical T‐cell exhaustion signature and its connection to T‐cell activation [4]. Furthermore, single‐cell analysis of infiltrating lymphocytes allows a detailed understanding of the role of these cells in the highly complex TME.

Head and neck cancer is the sixth most frequent malignancy worldwide. The incidence of oral cancer accounts for approximately 28% of that for head and neck cancer, and oral squamous cell carcinoma (OSCC) accounts for over 90% of oral cancers, with over 350 000 new cases and 170 000 deaths annually worldwide [5, 6]. The histological grade of OSCC can vary from well‐differentiated keratinizing to undifferentiated nonkeratinizing carcinoma, with a high tendency to metastasize. OSCC risk factors include alcohol consumption, smoking, betel nut chewing, viral infections (Epstein–Barr virus, human papillomavirus, and herpes simplex virus), occupational exposure to carcinogens, immunodeficiency, irradiation, and genetic predisposition [7, 8]. Furthermore, owing to demographic factors, the morbidity and mortality of OSCC are unevenly distributed [9]. For instance, OSCC is more prevalent in men than in women. Recently, the microenvironment of solid tumors has become a promising target for immunomodulating therapies, and immunotherapy with checkpoint inhibitors such as antiprogrammed death protein 1 (PD‐1) has shown promising results in treating patients with recurrent/metastatic OSCC. However, only a few patients benefit from tumor immunotherapy, partly owing to the high heterogeneity of tumor‐infiltrating lymphocytes. Systematic interrogation of tumor‐infiltrating lymphocytes is key to the development of immunotherapies and the ability to predict clinical responses. Therefore, we aimed to characterize the TME‐infiltrating lymphocytes in OSCC.

Although tumors are recognized as antigens by host T cells, the occurrence of immune escape prevents their clearance. This is partly due to the immunosuppression of the tumor microenvironment, mediated by PDCD1/PD‐ligand 1 (L1) upregulation. PDL1 expression contributes to the development of an immunosuppressive tumor milieu [10] and has been demonstrated in situ in a variety of solid tumors, including malignancies of the lung, bladder, liver, salivary gland, thyroid, thymus, head, and neck [11, 12, 13, 14]. Furthermore, the PDCD1/PDL1 interaction plays an important and diverse role in T‐cell activation, tolerance, and immune‐mediated tissue damage and tumorigenesis [10]. The overexpression of PDL1, triggered by interferon‐gamma (IFN‐γ) released by activated CD8^+^ T cells in the TME [15], induces T‐cell anergy and apoptosis by interacting with PDCD1, an immune checkpoint (IC) inhibitor expressed on the surface of immune cells [15, 16]. Considering the profound roles of PDCD1/PDL1, cancer immunotherapy based on PDCD1/PDL1 checkpoint blockade has been developed to reinvigorate CD8^+^ T cells in advanced or refractory cancers [16, 17, 18].

In this study, we performed scRNA‐seq of 11 866 single T cells isolated from the tumor and paired adjacent normal tissues of three patients with OSCC. We identified 14 unique T‐cell subsets in the tumor and five subclusters in normal tissue, indicating that the T‐cell population of the TME is more heterogeneous compared with that of normal tissue. Our scRNA‐seq data of infiltrating T cells in OSCC constitutes a valuable resource that enriches our understanding of the function of these cells in OSCC and can potentially lead to effective immunotherapy strategies.

## 2 Materials and methods

### 2.1 Human specimens

**Fig. 1:**
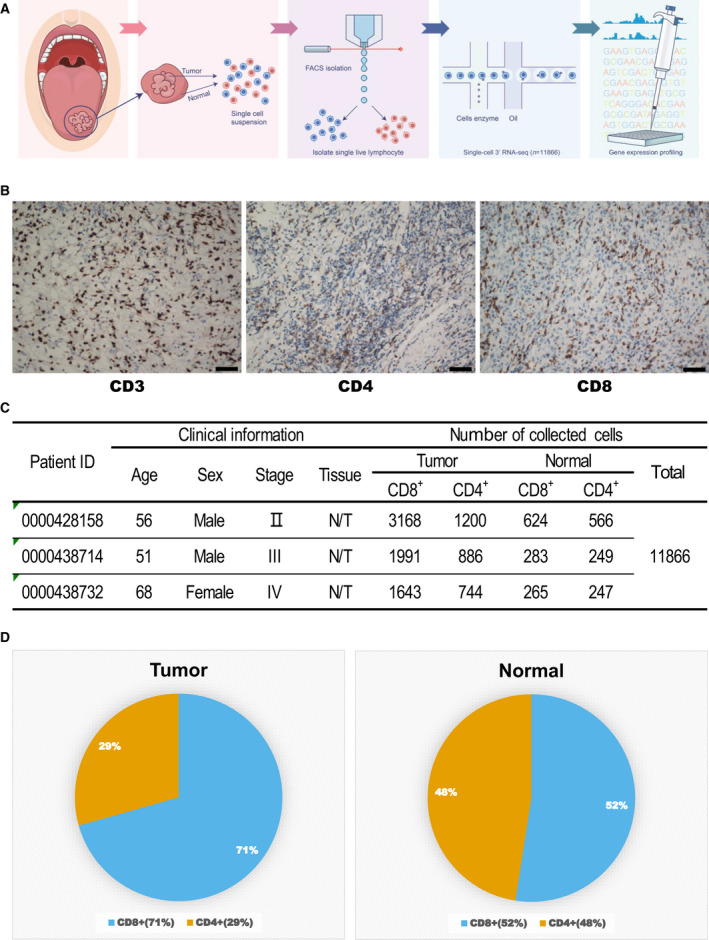
Overall characteristics of oral squamous cell carcinoma (OSCC) samples used for single‐cell RNA sequencing (scRNA‐seq). (A) Workflow for collection and processing of fresh biopsy samples of primary oral squamous cell carcinoma and matched adjacent normal tissues for scRNA‐seq. (B) Immunohistochemical staining using anti‐CD3, CD4, and CD8 antibodies. Scale bars, 100 μm. (C) Clinical characteristics of patients with OSCC and number of T cells sequenced. N/T represents T cells isolated from adjacent normal and tumor tissues. (D) The proportion of CD4^+^ and CD8^+^ cells in the tumor and normal tissues.

This study included one female and two male patients pathologically diagnosed with OSCC. The available clinical characteristics are summarized in Fig. 1C. Fresh OSCC tumor and paired adjacent normal tissues were obtained from the patients for subsequent lymphocyte isolation. The adjacent normal tissues were at least 2 cm away from the tumor tissues. Since all the specimens used were anonymous, the Medical Ethics Committee exempted patients from the need for informed consent and approved the study duly conforming the guidelines set by the Declaration of Helsinki

### 2.2 Cell lines

The OSCC cell lines (HSC‐3 and CAL‐33; both derived from male patients) used in this study were generously provided by Dr. Xiaoyan Fu and colleagues following cell‐line confirmation using short tandem repeat (STR) analysis (data not shown). HSC‐3 and CAL‐33 cells were grown in DMEM supplemented with 10% fetal bovine serum and 1X penicillin–streptomycin–glutamine.

### 2.3 Single‐cell collection and cDNA amplification

Single‐cell capture was performed using the Chromium Single Cell Controller (10x Genomics) (https://www.10xgenomics.com/solutions/single‐cell/). Fresh tissue was dissected and homogenized using tissue dissociation kits (Miltenyi Biotec, Bergisch Gladsbach, Germany). Single‐cell suspensions were then filtered through a 70‐μm nylon mesh filter (BD Biosciences, San Jose, CA, USA) in phosphate‐buffered saline (PBS) supplemented with 0.04% bovine serum albumin (BSA). Red blood cells were hypotonic lysed. Fresh cells were harvested, washed with 1× PBS, and resuspended at a density of 1× 10^6^ cells·mL^−1^ in 1× PBS containing 0.04% BSA to minimize cell loss and aggregation following the 10x Genomics protocol. Cell viability was analyzed using trypan blue exclusion staining. The cellular suspensions were loaded on a Chromium Single Cell Controller to generate single‐cell gel bead‐in‐emulsions (GEMs) using chromium single‐cell 3 reagent v3 kits (10x Genomics), containing a pool of ~750 000 barcodes sampled to separately index the transcriptome of each cell. Thousands of individual cells were isolated into droplets together with gel beads coated with unique primers bearing 10X cell barcodes, unique molecular identifiers (UMI), and poly (dT) sequences. GEM‐reverse transcriptions were performed using a Veriti 96‐well thermal cycler (ThermoFisher Scientific, Waltham, MA, USA), following the single‐cell 3 reagent kit protocol. Subsequently, the GEMs were broken, and the barcoded single‐strand cDNA was cleaned using DynaBeads MyOne Silane beads (ThermoFisher Scientific) and the SPRI Select Reagent Kit (Beckman Coulter, Fullerton, CA, USA). Global amplification of cDNA was achieved using the Veriti 96‐well thermal cycler, and the amplified cDNA product was cleaned using the SPRI Select Reagent Kit.

### 2.4 Library construction and sequencing

The indexed sequencing libraries were constructed using the reagents in the Chromium Single Cell 3 Library v3 Kit for: (a) fragmentation, end repair, and A‐tailing; (b) size selection using SPRI select beads; (c) adaptor ligation; (d) postligation cleanup using SPRI select beads; and (e) sample index PCR and final cleanup using SPRI select beads. The final single‐cell 3 library comprises the standard Illumina paired‐end constructs, which begin and end with the P5 and P7 primers used in Illumina bridge amplification. The barcoded sequencing libraries were quantified using a Bioanalyzer Agilent 2100 System, a High Sensitivity DNA Chip (Agilent Technologies, Palo Alto, CA, USA), and quantitative PCR using a KAPA Library Quantification Kit (KAPA Biosystems, Wilmington, MA, USA). Finally, the sequencing libraries were loaded onto a NovaSeq 6000 (Illumina, San Diego, CA, USA) with paired‐end sequencing mode.

### 2.5 Data quality and filtering

Raw reads were aligned to the GRCh38 (release 93) human reference genome to obtain the raw count matrix using the Cellranger toolkit (v3.0.2). In the raw count matrix, genes expressed in fewer than three cells were discarded. Cells with fewer than 200 genes or over 20% mitochondrial gene expression were considered as low‐quality cells. Cells with over 6000 genes were considered as doublets. Low‐quality cells and doublets were excluded from further analyses.

### 2.6 Cluster and pseudotime analysis

Cluster analysis of the single‐cell count matrices was performed using the R package ‘Seurat’ (v3.1.0) [19]. In brief, normalization and scaling were performed after filtering using the ‘NormalizationData’ and ‘ScaleData’ functions with the default parameters. The principal components for highly variable genes were calculated using the ‘RunPCA’ function. Based on the elbow plot of the principal component analysis, the 30 most significant components were used to identify clusters using the ‘FindClusters’ function with a 0.5 resolution. T‐distributed stochastic neighbor embedding (T‐SNE) was used to visualize the clusters in a reduced 2D space using the ‘RunTSNE’ and ‘TSNEPlot’ functions. Finally, the ‘FindAllMarkers’ function was executed to find the cluster markers. Cluster cell types were assigned according to cluster markers and cluster labels from the R package ‘SingleR’ (v1.0.1) [20]. Furthermore, clusters were characterized using the ‘DoHeatmap’ function while pseudotime analysis was performed using the R package ‘monocle 2’ (v2.12.0) [21, 22]. Briefly, matrices used in monocle 2 were converted from the ‘Seurat’ objects using the ‘importCDS’ function. Genes for ordering were selected from those that differed between the clusters defined in the cluster analysis. Then, the dimensions were reduced, and the cells ordered using the ‘reduceDimension’ and ‘orderCells’ functions with the default parameters. Finally, the ‘plot_cell_trajectory’ function was used to visualize the cells in trajectory.

### 2.7 Gene ontology enrichment analysis

The gene ontology (GO) characteristics of the clusters were determined using the R package ‘clusterProfiler’ (v3.12.0) [23]. Genes with an average logFC value above zero and an adjusted *P* value below 0.05 were used for enrichment. To compare the enrichments between different clusters, the ‘compareCluster’ function was executed on these selected genes using a 0.05 *P* value cutoff.

### 2.8 Data availability statement

The raw scRNA‐seq data were deposited in the NCBI Sequence Read Archive (SRA) database (BioProject accession ID: PRJNA650256). The authenticity of this manuscript has been validated by uploading the key raw data to the Research Data Deposit (RDD) public platform (www.researchdata.org.cn) under the approval RDD number RDDB2020000940.

### 2.9 Isolation and activation of human T cells

Human peripheral blood mononuclear cells (PBMCs) were harvested and then CD8^+^ T cells sorted using CD8 magnetic microbeads. CD8^+^ T cells were then activated using Dynabeads Human T‐Activator CD3/CD28 at a ratio of 1 : 1 for 48 h.

### 2.10 Overexpressing PDCD1 and thymocyte selection‐associated high‐mobility group box (TOX) in activated CD8^+^ T cells

Human PDCD1 or TOX was cloned into the retroviral vector MSCV‐IRES‐EGFP (RV‐Vec). The retrovirus was generated using the pAmpho packaging system. For T‐cell infection, activated CD8^+^ T cells were spin‐infected with the retrovirus at 2000 rpm for 1 h at 32°C. Three days postinfection, GFP^+^ human PDCD1‐ or TOX‐overexpressing (OE) CD8^+^ T cells were sorted using fluorescence‐activated cell sorting (FACS).

### 2.11 Coculture and apoptosis assays

PDL1^+^ HSC‐3 and PDL1^+^ CAL‐33 cells were FACS‐sorted and then cocultured with nonspecific control (Ctrl) OE‐activated CD8^+^ T cells, activated human PDCD1 OE CD8^+^ T cells, activated human TOX OE CD8^+^ T cells, or activated human TOX OE CD8^+^ T cells with anti‐PD1 (aPD1). Tumor cells were collected at 4, 8, and 12 h. Apoptosis assays were performed using the Dead Cell Apoptosis Kit with Annexin V‐FITC and propidium iodide, according to the manufacturer’s protocol, using a BD FACS Calibur flow cytometer.

### 2.12 Carboxyfluorescein succinimidyl amino ester (CFSE) labeling

Three types of human CD8^+^ T cells (Ctrl OE CD8^+^ T cells, PDCD1 OE CD8^+^ T cells, and TOX OE CD8^+^ T cells) were cultured in T‐cell culture medium (IMDM plus 10% fetal bovine serum) under CD3/CD28 stimulation. T cells were labeled with CFSE, collected on day 2, and then their proliferating function was measured using a flow cytometer.

### 2.13 Dual‐luciferase reporter assays

The human *TOX* promoter (from −2000 to +100 bp) was synthesized and subcloned into the pEZX‐FR01 vectors (GeneCopoeia). For *TOX* promoter activity, Jurkat cells were electronically transfected with reporter plasmids as well as plasmids overexpressing PRDM1. Dual‐luciferase reporter assays were performed 48h after transfection with the Luc‐Pair ™ Duo‐Luciferase HS Assay Kit (GeneCopoeia, LF004) according to the manufacturer’s instructions. Firefly luciferase activity was normalized to Renilla luciferase activity for each sample.

## 3 Results

### 3.1 Tumor characteristics and single T‐cell transcriptome data generation

To investigate the complexity of infiltrating T cells in OSCC, we performed scRNA‐seq on FACS‐sorted T cells isolated from tumors and adjacent normal tissues from three patients with OSCC (Fig. 1A). After confirming the presence of infiltrating lymphocytes using immunohistochemical staining (IHC) for CD3, CD4, and CD8 (Fig. 1B), consistent with previous reports [11], we sorted Calcein^+^ CD3^+^ living infiltrating T cells from single‐cell suspensions of tumors and adjacent normal tissues (Fig. S1) and obtained single‐cell transcriptome data from a total of 11,866 cells (Fig. 1C). Interestingly, the percentage of CD8^+^ T cells was much higher in tumor tissue than that in paired adjacent normal tissue (70.6% vs. 52.4%, Fig. 1C,D), indicating a potential enrichment of CD8^+^ cytotoxic T cells in the TME due to tumor antigen stimulation. The CD4:CD8 ratio was decreased (0.91 vs. 0.42), indicating a more robust antigen‐experienced antitumor immune response [24]. Tumor progression was therefore indicated based on enrichment of CD8^+^ cytotoxic T cells in the TME and the molecular components and functional difference between the tumor‐infiltrating T cells and normal tissue‐resident T cells. This highlighted the need for single‐cell technologies that allow a more detailed dissection of tumor‐associated T cells in an unbiased and high‐throughput manner.

### 3.2 T‐cell clustering and subtype analysis of the TME

**Fig. 2:**
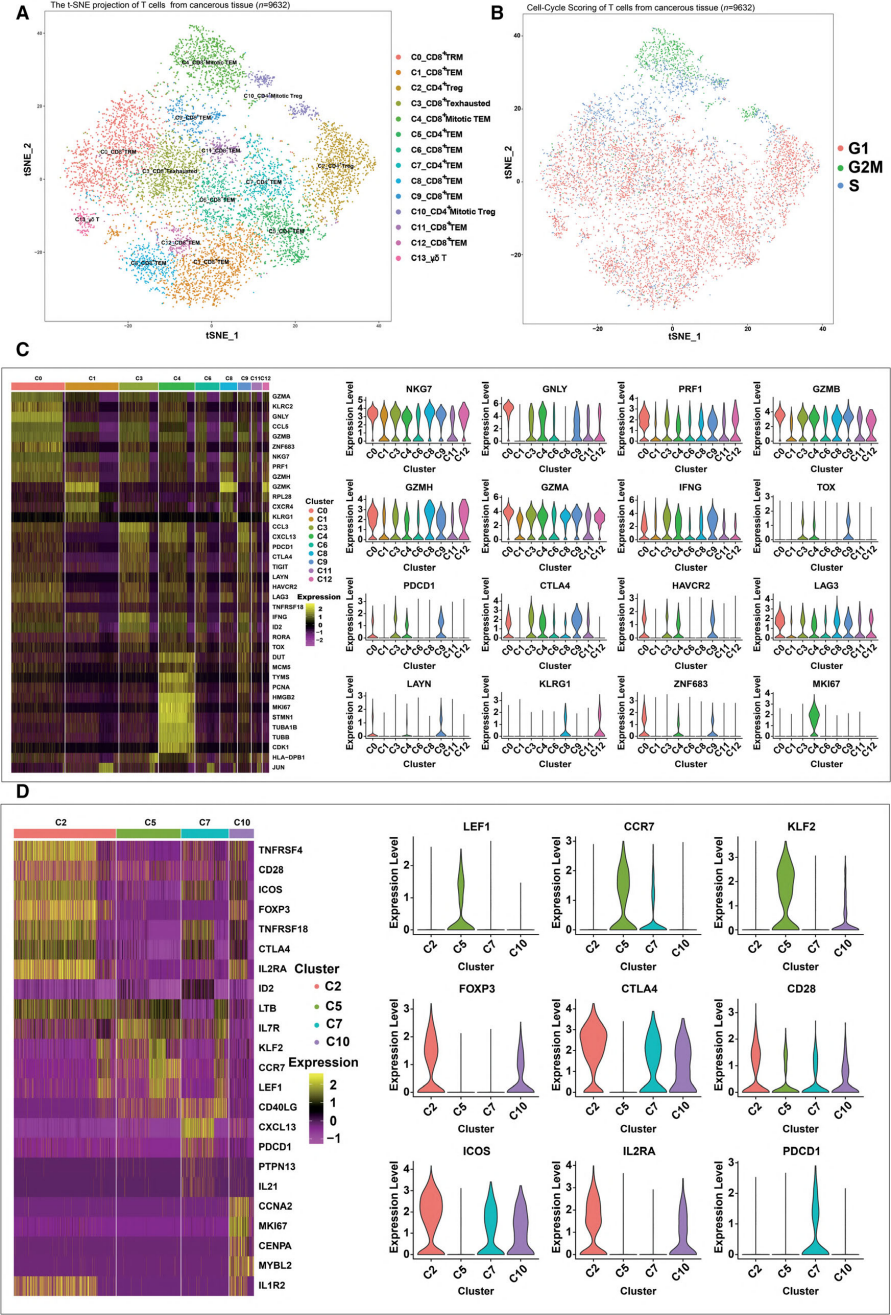
Expression heterogeneity of CD8^+^ and CD4^+^ T cells in the OSCC ecosystem. (A) The t‐distributed stochastic neighbor embedding (t‐SNE) projection of T cells from cancerous tissue of patients with OSCC, showing the formation of 11 main clusters in different colors. The functional description of each cluster is determined by the gene expression characteristics. Cluster 0: resident memory CD8^+^ T Cells (C0_CD8^+^ TRM); Cluster 1: effector memory CD8^+^ T cells (C1_CD8^+^ TEM); Cluster 2: regulatory CD4^+^ T cells (C2_CD4^+^ Treg); Cluster 3: exhausted CD8^+^ T cells (C3_CD8^+^ T Exhausted); Cluster 4: effector memory M‐phage CD8^+^ T cells (C4_CD8^+^ Mitotic TEM); Cluster 5: effector memory CD4^+^ T cells (C5_CD4^+^ TEM); Cluster 6: effector memory CD8^+^ T cells (C6_CD8^+^ TEM); Cluster 7: effector memory CD4+ T cells (C7_CD4^+^ TEM); Cluster 8: effector memory CD8^+^ T cells (C8_CD8^+^ TEM); Cluster 9: effector memory CD8^+^ T cells (C9_CD8^+^ TEM); Cluster 10: effector memory M‐phage CD4^+^ T cells (C10_CD4^+^ Mitotic TEM); Cluster 11: effector memory CD8^+^ T cells (C11_CD8^+^ TEM); Cluster 12: effector memory CD8^+^ T cells (C12_CD8^+^ TEM); Cluster 13: γδ T. (B) Cell‐Cycle Scoring of T cells from cancerous tissue of patients with OSCC, showing the three clusters of cells in different cell cycles in different colors. (C) Heatmap of key genes expressed in each cluster of CD8^+^ tumor‐infiltrating lymphocytes (TILs) with cells grouped by clusters. The columns correspond to the cells; the rows correspond to the genes. Yellow: high expression; purple: low expression. Selected key markers are shown on the right. (D) Heatmap of key genes expressed in each cluster of CD4^+^ TILs with cells grouped by clusters. The columns correspond to the cells; the rows correspond to the genes. Yellow: high expression; purple: low expression. Selected key genes are shown on the right.

Based on the gene expression profiles, we identified 14 distinct cell clusters in the tumors, including nine CD8^+^ T‐cell clusters (0, 1, 3, 4, 6, 8, 9, 11, and 12), four CD4^+^ T‐cell clusters (2, 5, 7, and 10), and one γδ T‐cell cluster (13), each with unique signature functions (Fig. 2A and S2A). The clusters were identified according to their marker gene expression (Fig. S2B and S2C). Among the different CD8^+^ clusters, cluster 0 comprised tissue‐resident memory T cells (T_RM_) owing to a high expression of ZNF683 [25, 26] (Fig. 2B,C). This T_RM_‐like cluster exhibited a high expression from genes of effector markers such as NKG7, GNLY, PRF1 (Perforin, GZMB (Granzyme B), GZMH, and GZMA (Fig. 2B,C), which linked the T_RM_ signature to the magnitude of the cytotoxic T‐cell response [27]. However, this cluster also showed high gene expression of immune checkpoints, including CTLA4, HAVCR2 (TIM3), and LAG3 (Fig. 2B,C), which was consistent with the results of recent studies [28]. Cluster 1 was characterized as effector memory T cells (T_EM_) by expression of genes for NKG7, GZMB, GZMH, and GZMA, which linked the T_EM_ signature to the effector memory [27]. Cluster 3 showed a high expression genes of checkpoint markers, including PDCD1, CTLA4, TIGIT, HAVCR2 (TIM3), LAG3, and TNFRSF18 (Fig. 2B,C). Based on our data and those of previous studies [29, 30, 31], these markers were also highly expressed in exhausted T cells (Fig. 2C); thus, cluster 3 was defined as the exhausted T‐cell (T_EX_) cluster, suggesting that this might play an important role in T‐cell exhaustion in OSCC. Cluster 4 showed high expression of cell cycle‐related genes for markers such as PCNA, MCM5, MKI67, STMN1, and CDK1 (Fig. 2B,C), probably owing to the immune response to cancer neo‐epitopes. Cluster 4 was defined as the mitotic T_EM_ cluster, based on CellCycleScoring (Fig. 2C). Although this cluster exhibited high *IFNG* expression, it also expressed a high level of checkpoint molecules, as did Cluster 2 (Fig. 2B,D), confirming that the CD8^+^ exhausted T cells in OSCC were heterogeneous and belonged to distinct subsets [32, 33]. Five clusters, 6, 8, 9, 11, and 12, were characterized as T_EM_ clusters owing to the high expression of effector memory marker genes for markers such as NKG7, PRF1, GZMB, GZMH, GZMA, and IFNG [27, 34]. In addition, clusters 8 and 12 showed specific expression of the late‐differentiated effector and effector memory marker KLRG1 [35, 36] (Fig. 2B,C). Cluster 13 was designated as the γδ T‐cell cluster for expression of genes for the relevant markers TRDC, XCL1, and XCL2 [28].

Similarly, we identified four major CD4^+^ T‐cell clusters (Fig. S2C). Cluster 2, comprising the majority of CD4^+^ T cells, was identified as the regulatory T‐cell (Treg) cluster owing to the specific mRNA expression of the well‐defined Treg marker *FOXP3* [37] (Fig. 2D). Interestingly, this cluster exhibited high expression from genes of costimulatory molecules, such as CD28 and ICOS, in addition to other well‐defined Treg markers, such as CTLA4 [38, 39] (Fig. 2D), indicating the complex role of Tregs in immunomodulation. Compared with cluster 2, cluster 10, defined as the mitotic Treg cluster, exhibited a similar gene expression profile except for the high expression of genes for proliferation‐associated proteins such as CCNA2 and MKI67 (Fig. 2D). Cluster 5 expressed high levels of ‘naïve’ markers such as LEF1, CCR7, and KLF2 [40] (Fig. 2D), thereby representing a group of less differentiated effector memory T cells. However, Cluster 7 expressed high levels of *PDCD1* and *CTLA4* mRNA (Fig. 2D), which was suggestive of exhausted CD4^+^ T cells.

### 3.3 Pseudotime analysis revealed the dynamic state transitions of OSCC‐infiltrating lymphocytes

The scRNA‐seq transcriptome data for thousands of tumor‐infiltrating T cells provided insight into the functional states and relationships between the distinct T‐cell subsets. We applied the Monocle 2 algorithm [21] to construct the potential developmental trajectories of CD8^+^ and CD4^+^ clusters.

**Fig. 3:**
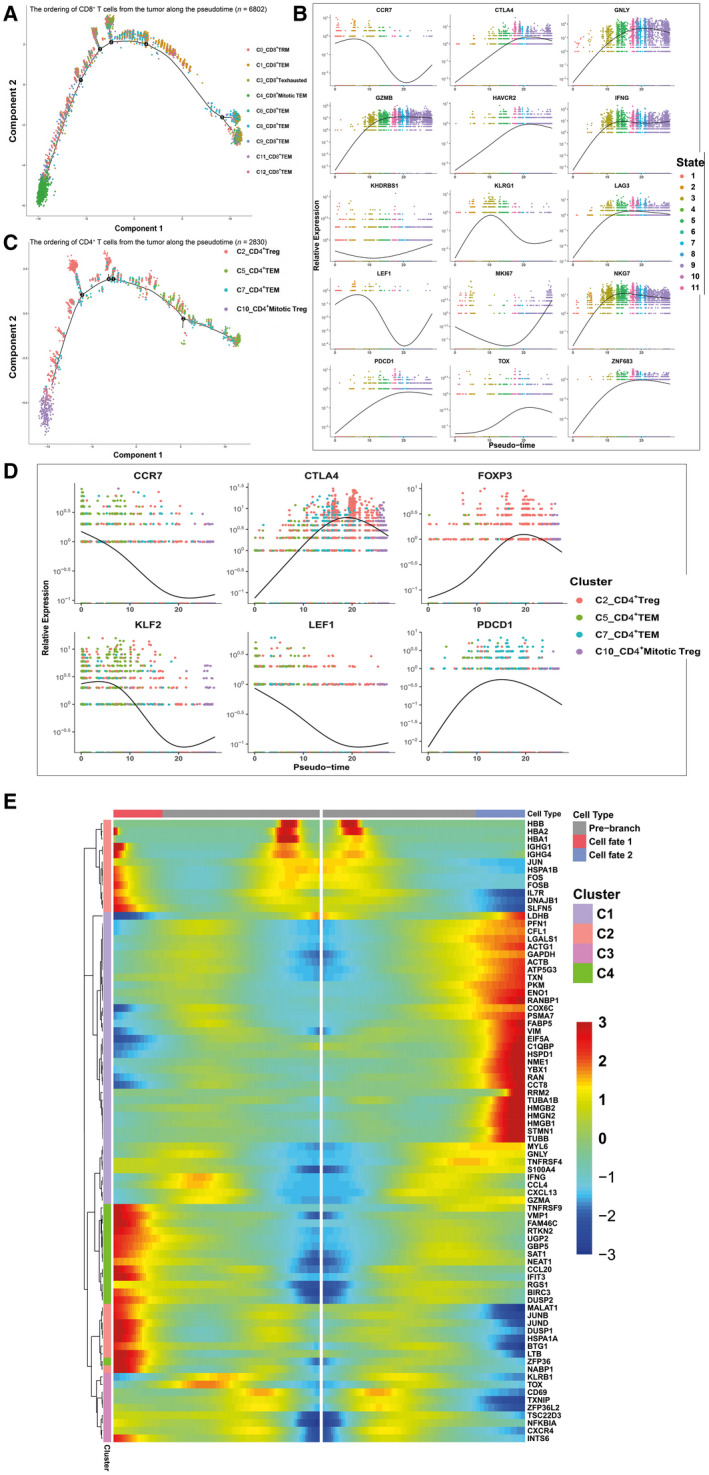
Gene expression dynamics along the pseudotime of T‐cell development. (A) The ordering of CD8^+^ T cells from the tumor along the pseudotime in a two‐dimensional state‐space. Each point corresponds to a single cell, and each color represents a T‐cell cluster. (B) The ordering of the different marker genes of CD8^+^ T cells from the tumor along the pseudotime in a two‐dimensional state‐space. Each point corresponds to a single cell, and each color represents a T‐cell cluster. A natural spline was used to model gene expression as a smooth, nonlinear function over the pseudotime. (C) The ordering of CD4^+^ T cells from the tumor along the pseudotime in a two‐dimensional state‐space. Each point corresponds to a single cell, and each color represents a T‐cell cluster. (D) The ordering of the different marker genes of CD4^+^ T cells from the tumor along the pseudotime in a two‐dimensional state‐space. Each point corresponds to a single cell, and each color represents a T‐cell cluster. A natural spline was used to model gene expression as a smooth, nonlinear function over the pseudotime. (E) Heatmap depicting genes in a branch‐dependent manner for branch point 4. Each row represents the dynamic expression of a gene.

Regarding the trajectory of CD8^+^ clusters (Fig. 3A and S3A), the lack of branching reflected the absence of distinct precursors as well as the terminally differentiated nature of the CD8^+^ T cells infiltrating the TME. Three clusters, 4_CD8^+^ mitotic T_EM_, 0_CD8^+^ T_RM_, and 9_CD8^+^ T_EM_, were positioned at the opposite end of cluster 6_CD8^+^ T_EM_ and 11_CD8^+^ T_EM_ while clusters 1_CD8^+^ T_EM_, 2_CD8^+^ T_EX_, 8_CD8^+^ T_EM_, and 12_CD8^+^ T_EM_ were located in between these, indicating their intermediate differentiation states. Notably, clusters 8_CD8^+^ T_EM_ and 3_CD8^+^ T_EX_ were located at adjacent positions, suggesting that in OSCC, these two tesla cell subgroups are highly heterogeneous and that they should not be viewed as a homogeneous population. Consistent with our data, a recent study implied that only partially resembled that seen in chronic infection, tumor reactivity was still largely restricted to the PD‐1‐high lymphocytes in non‐small‐cell lung cancer [41], indicating the high complexity of T‐cell exhaustion in the OSCC microenvironment. Three proliferative clusters, 0_CD8^+^ T_RM_, 4_CD8^+^ mitotic T_EM_, and 9_CD8^+^ T_EM_, were located at opposite ends of the branch of the pseudotime path, indicating their distinct gene expression profiles compared with that of other clusters. We then used well‐known cluster markers such as CCR7, LEF1, PDCD1, HAVCR2, LAG3, CTLA4, IFNG, KLRG1, ZNF683, GZMB, NKG7, GNLY, and MKI67 to demonstrate the dynamic changes of the relevant genes in pseudotime (Fig. 3B). We found that during T‐cell differentiation, mRNA expression levels of naïve related markers (CCR7 and LEF1) decreased dramatically while those of exhausted markers (CTLA4, HAVCR2, LAG3, and PDCD1) and effector molecules (GNLY, GZMB, IFNG, and NKG7) increased gradually but dropped at the later mitotic phase, with expression of mitotic genes (*MKI67* and *KHDRBS1*) peaking at the mitotic phase.

Similarly, we analyzed CD4^+^ T cells to determine their differentiation trajectory, which consisted of four branches (Fig. 3C). Cluster 5_CD4^+^T_EM_, which expressed high levels of naïve marker mRNA, was situated at the starting point. The pseudotime trajectory evolved into two directions, one toward cluster 7_CD4^+^ T_EM_ and another toward cluster 2_CD4^+^ Treg. Similar to CD8^+^ T cells, mitotic Tregs were positioned at the opposite end. As CD4^+^ T lymphocytes differentiated, T cells transitioned from the naïve state to branch point 4, then to the regulatory or exhausted state, representing the procedure of CD4^+^ T‐cell differentiation from naïve T cells to Treg or T_EX_, during which expression of naïve marker mRNA gradually decreased while that of Treg marker mRNA dramatically increased (Fig. 3D). Furthermore, we identified branch‐dependent genes for branch point 4 (Fig. 3E). Cells transitioning from state 3 to state 6 had high mRNA expression of Treg‐associated proteins (FOPX3, CTLA4, TNFRSF18, and TNFRSF4), whereas the mRNA expression of effector molecules (GZMA, GNLY, IFNG, GZMB, and NKG7) and chemokines (CCL3, CCL4, and CCL5) increased when cells transitioned to state 5.

In summary, the results of quasi‐temporal analysis for CD8^+^ and CD4^+^ T cells are consistent with those of clustering and subtype analysis and those of Puram *et al*. [42]. The expression states of these T cells may contribute to understanding and predicting responses to checkpoint immunotherapies [43].

### 3.4 Functional heterogeneity and prognostic values of T‐cell clusters in patients with OSCC

**Fig. 4:**
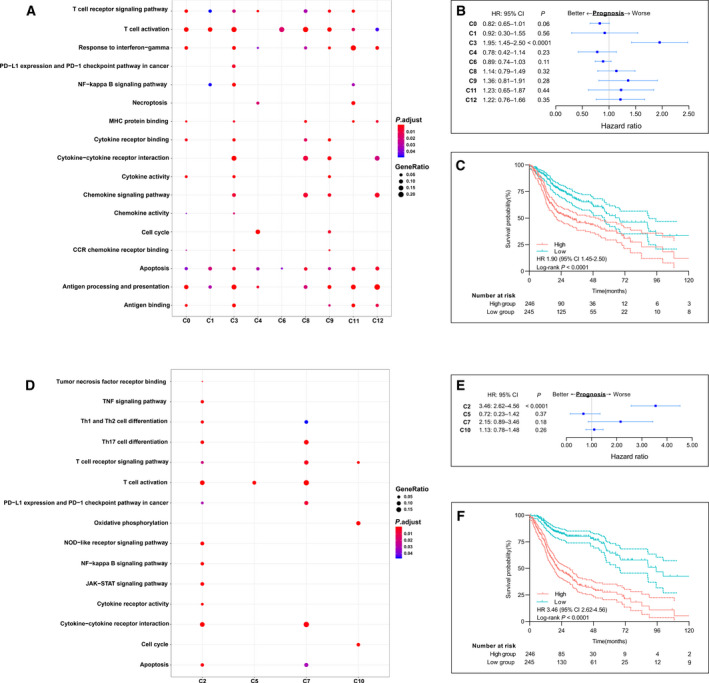
Prognostic values of immune signatures of each cluster of T cells in patients with OSCC. (A) Gene Ontology (GO) Biological Process enrichment plot for clusters of CD8^+^ T cells in tumors. (B) Forest plots show hazard ratios (HRs; blue squares), confidence intervals (CIs, horizontal ranges), and *P* values of each signature gene cluster of CD8^+^ T cells; cluster 3 has a clear prognostic value (HR: 1.95, 95% CI: 1.45–2.50, log‐rank *P* < 0.0001). (C) Survival curves of The Cancer Genome Atlas (TCGA) cohorts of patients with head and neck squamous cell carcinoma (HNSCC). Patients were grouped according to the level of cluster 3 gene signature derived from single‐cell data (HR: 1.95, 95% CI: 1.45‐2.50, log‐rank *P* <0.0001). (D) GO enrichment plots for clusters of CD4^+^ T cells in tumor. (E) Forest plots show HRs (blue squares), CIs (horizontal ranges) and *P* values of each cluster signature genes of CD4^+^ T cells; cluster 2 has a clear prognostic value (HR: 3.46, 95% CI: 2.62–4.56, log‐rank *P *< 0.0001). (F) Survival curves of TCGA cohorts of patients with head and neck squamous cell carcinoma (HNSCC). Patients were grouped according to the level of cluster 2 gene signature derived from single‐cell data (HR: 3.46, 95% CI: 2.62–4.56, log‐rank *P *<0.0001).

To further understand the functional heterogeneity of OSCC‐infiltrating lymphocytes, the differentially expressed marker genes of the CD8^+^ and CD4^+^ clusters were assessed via GO analysis [23]. The significantly enriched GO terms of tumor CD8^+^ T cells included ‘cell cycle’, ‘T cell activation’, ‘NF‐κB signaling pathway’, ‘PD‐L1 expression and PD‐1 checkpoint pathway in cancer’, ‘response to interferon‐gamma’, ‘apoptosis’ [44], ‘antigen processing and presentation’ (Fig. 4A), showing divergent functional specialization. Consistent with the T‐cell clustering and subtype analysis results shown in Fig. 2, the highly proliferative cluster 4_CD8^+^ mitotic T_EM_ was highly enriched for cell cycle processes. The ‘PD‐L1 expression and PD‐1 checkpoint pathway in cancer’ processes were specifically enriched in cluster 3_CD8^+^ T_EX_, which was previously identified as comprising exhausted T cells. Although ex vivo reactivation experiments have demonstrated that CD8^+^ exhausted T cells produce fewer effector cytokines [42], our analyses revealed that cluster 3_T_EX_ was also enriched for pathways related to T‐cell activation and function, indicating that the T_EX_ cells may not have completely lost their antitumor effector potential in vivo [33]. To further understand the phenotype of this cluster, we analyzed the independent head and neck squamous cell carcinoma (HNSCC) cohort from The Cancer Genome Atlas (TCGA). The results indicated that cluster 3 has prognostic value [HR (95% CI): 1.95 (1.45–2.50), log‐rank *P *< 0.0001, Fig. 4B], and patients with low expression of the signature genes of cluster 3 showed significantly better overall survival compared with that of patients with high expression of these genes [HR (95% CI): 1.95 (1.45–2.50), log‐rank *P* <0.0001, Fig. 4C, Table S1A]. Furthermore, cluster 0_CD8^+^ T_RM_ was prominently enriched for immune‐related pathways (Fig. S4A), suggesting a special role of tissue‐resident memory T cells in the control of solid tumors and their potential applications for treating patients with cancer [45]. Surprisingly, the apoptosis pathway was enriched in all clusters, suggesting that tumor‐infiltrating T cells underwent a vicious cycle of activation and death that restrained the number of T cells within the TME, leading to inadequate tumor control, as was observed in a B16.SIY murine melanoma model [46].

Regarding CD4^+^ T cells, distinct GO terms were enriched, including ‘JAK‐STAT signaling pathway’, ‘Th17 cell differentiation’, ‘NOD‐like receptor signaling pathway’, ‘Th1 and Th2 differentiation’, and ‘TNF signaling pathway’ (Fig. 4D). Focusing on pathways related to T‐cell activation and function, cluster 2_CD4^+^ Treg cells were enriched in almost all the associated pathways, including ‘JAK‐STAT signaling pathway’, ‘Th17 cell differentiation’, ‘Th1 and Th2 differentiation’, ‘T cell activation’, ‘T cell receptor signaling pathway’ and ‘NF‐kappa B signaling pathways’ indicating that Tregs are a subpopulation of functionally active effector cells (Fig. 4D and S4B). To understand the complex role of cluster 2 in the TME, using the available gene expression data from the cohort from TCGA, we found that cluster 2 had a prognostic value [HR (95%CI): 3.46 (2.62–4.56), log‐rank *P* <0.0001, Fig. 4E] and that low expression of Treg was significantly associated with better overall survival [HR (95%CI): 3.46 (2.62–4.56), log‐rank *P* <0.0001, Fig. 4F, Table S1B]. CD4^+^ Treg cells were the dominant subset of CD4^+^ T helper (Th) cells as this was found in a greater quantity (Fig. S6A) and in an active state. Metabolism plays a critical role in T‐cell function, differentiation, and longevity [47]. Tregs exhibit a high level of oxidative phosphorylation pathway activity, which may promote Treg differentiation and survival in the TME [48, 49]. Although a recent study indicated that promoting fatty acid catabolism in CD8^+^ tumor‐infiltrating T cells enhances their antitumor ability [50], in CD4^+^ Th cells may direct Treg formation, thus enhancing TME immunosuppression [51]. As mentioned above, cluster 10_Mitotic Treg was enriched for ‘cell cycle pathway’ while cluster 7_CD4^+^ T_EM_ was enriched for ‘PD‐L1 expression and PD‐1 checkpoint pathway in cancer’, indicating the phenotype of T‐cell exhaustion. Cluster 5_CD4^+^ T_EM_ exhibited the least enrichment for pathways related to T‐cell effector function except for ‘T cell activation’ signaling, which was consistent with the naïve state in pseudotime analysis.

In summary, the results of the GO enrichment term of T cells (Fig. 4 and S4) were consistent with the results of clustering (Fig. 2) and pseudotime analysis (Fig. 3), revealing the existence of OSCC‐infiltrating T‐cell subpopulations with high functional heterogeneity.

### 3.5 Normal tissue‐infiltrating T cells were more homogeneous

**Fig. 5:**
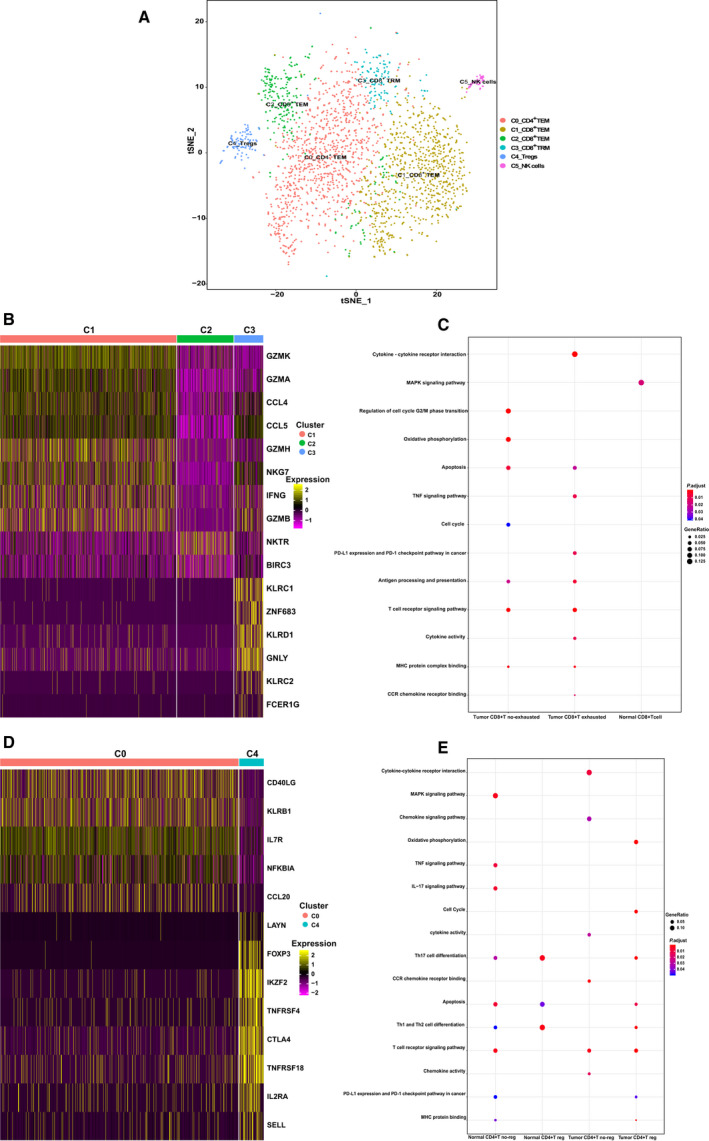
Heterogeneity of CD8^+^ and CD4^+^ T cells in normal tissue. (A) The t‐distributed stochastic neighbor embedding (t‐SNE) projection of T cells from adjacent normal tissue of patients with OSCC, showing the formation of six main clusters in different colors. The functional description of each cluster is determined by the gene expression characteristics. Cluster 0: C0_CD4^+^ T_EM_; Cluster 1: C1_CD8^+^ T_EM_; Cluster 2: C2_CD8^+^ T_EM_; Cluster 3: C3_CD8^+^ T_RM_; Cluster 4: C4_Tregs; Cluster 5: C5_NK cells. (B) Heatmap of key genes expressed in normal tissue CD8^+^ T‐cell clusters with cells grouped by clusters. Columns correspond to the cells, and rows correspond to the genes. Yellow: high expression; purple: low expression. Selected key genes are shown on the right. (C) Gene ontology (GO) enrichment plots for CD8^+^ T cells in the tumor and adjacent normal tissue. (D) Heatmap of normal CD4^+^ T cells, with two main clusters identified, each containing a unique set of signature genes. Selected genes with high expression are shown on the right. Yellow: high expression; red: low expression. (E) GO enrichment plots for CD4^+^ T cells in the tumor and adjacent normal tissue.

Compared with tumor‐infiltrating T lymphocytes, T cells infiltrating normal tissue were far less heterogeneous. Only three CD8^+^ clusters (1, 2, and 3), two CD4^+^ clusters (0 and 4), and a small cluster of natural killer (NK) T cells (FGFBP2+, cluster 6) were identified in normal tissue‐infiltrating T cells (Fig. 5A and S5). Cluster 3 was characterized as comprising tissue‐resident memory T (T_RM_) cells because of the specific expression of the T_RM_ marker ZNF683. Except for population decline (11.1% vs. 26%, Fig. S6A), cluster 3 expressed far fewer effector molecules than T_RM_ in the tumor, suggesting that in the TME, the stimulation of tumor neo‐epitopes led to the local activation and clonal expansion of T_RM_ in situ, thus suppressing cancer growth [52]. Cluster 1 was the major effector subset of CD8^+^ T cells in normal tissues because of the high expression levels of genes for effector molecules (GZMK, GZMA, GZMH, NKG7, IFNG, and GZMB) and chemokines (CCL4 and CCL5) (Fig. 5B), and the enrichment for pathways of T‐cell activation and function (Fig. S6B). In addition, we noticed that CD8^+^ proliferative and exhausted subpopulations were absent in normal tissue compared with those in the primary tumor (Fig. 5C and S5), suggesting a rather quiescent state of CD8^+^ T cells in normal tissue. Regarding CD4^+^ T cells, two clusters were characterized according to their marker genes (Fig. 5D), a small subset of T cells, cluster 4, was characterized as Tregs because of the specific expression of FOXP3. Tregs support tissue function and organismal homeostasis in normal tissue [53], and in the tongue primarily by means of highly expressed checkpoint molecules such as CTLA4, LAYN, TNFRSF4, and TNFRSF18. However, excessive accumulation of Tregs in the TME (9.3% vs. 43.0%, Fig. S6) may contribute to an immunosuppressive microenvironment [54]. Similar to CD8^+^ T_RM_, Tregs were more quiescent than their tumor counterparts, as they expressed low levels of costimulatory molecules and lower enrichment for the T‐cell activation pathway (Fig. 5D). Cluster 0 was identified by T_EM_ and found to be enriched for the TNF and NF‐kappa B signaling pathways using GO analysis (Fig. S6D).

The above findings revealed that normal tissue T cells differed from those in the primary tumor in cell composition and functional state, which implied that the TME was highly complex and heterogeneous.

### 3.6 PD1 and PDL1 were upregulated in OSCC

**Fig. 6:**
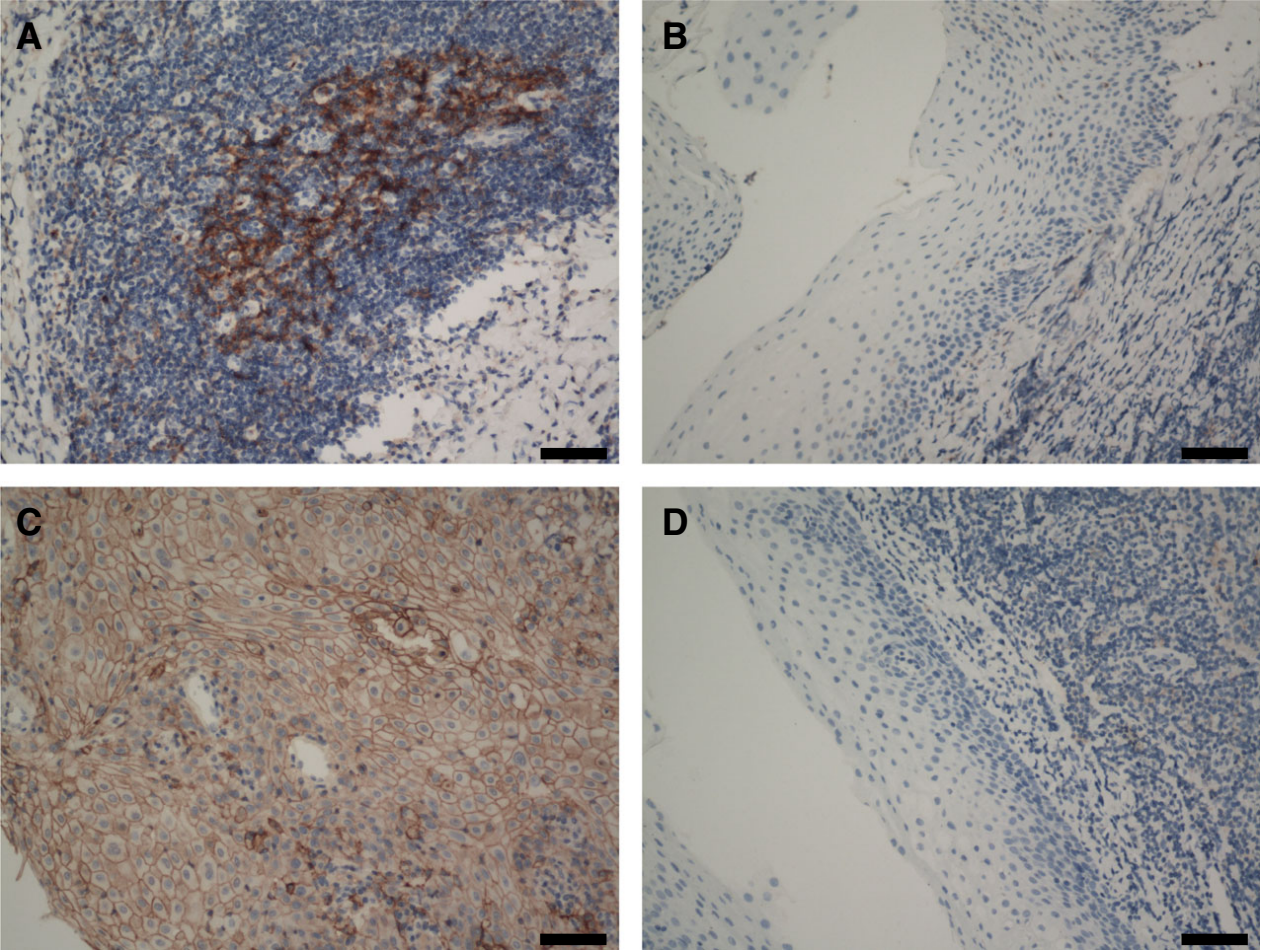
Results of immunohistochemical staining using antiprogrammed death protein‐1 (PD‐1) and PD‐ligand 1 (PD‐L1) antibodies (*n *= 30). Scale bars, 100 μm. (A) Positive staining for PD‐1 on tumor‐infiltrating lymphocytes. (B) Negative staining for PD‐1 on normal tissue‐infiltrating lymphocytes. (C) Positive staining for PD‐L1 on tumor cells. (D) Negative staining for PD‐L1 on normal cells.

We collected matched tumor and normal tissues from 30 OSCC patients and performed immunohistochemical staining on them. The IHC results of normal and malignant oral squamous epithelium are shown in Fig. 6. Expression of PD1 was upregulated in OSCC‐infiltrating lymphocytes (Fig. 6A), but not in lymphocytes infiltrating in normal tissue (Fig. 6B) while that of PDL1 was upregulated in OSCC tumor cells (Fig. 6C), but not in normal cells (Fig. 6D). Notably, lymphocytes were more abundant in cancer cell nests than in normal tissue areas. These results indicated that PD1^+^‐exhausted T cells are widespread in OSCC, which is consistent with the results of our scRNA‐seq data.

### 3.7 T‐cell phenotypes suggest regulators modulate the OSCC antitumor immune response

**Fig. 7:**
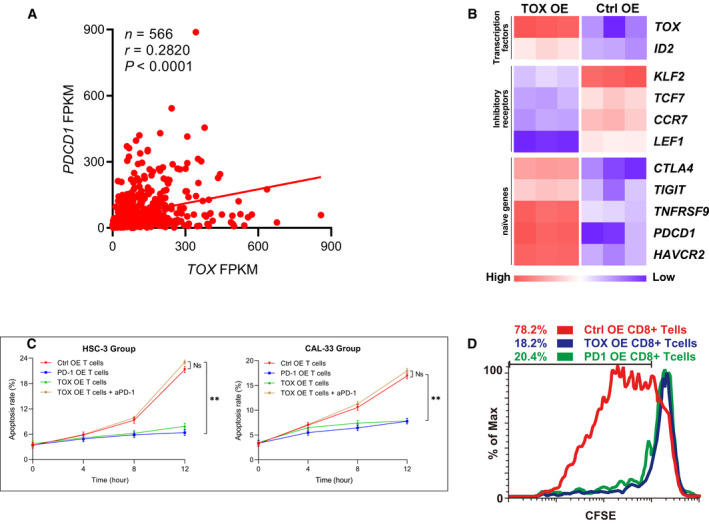
In vitro experiments on the effects of and thymocyte selection‐associated high‐mobility group box (TOX) on the antitumor function of human T cells. (A) Schematic of linear regression revealed a positive correlation between *TOX* expression level and (programmed death protein 1) *PDCD1* expression level in The Cancer Genome Atlas (TCGA) expression profiles (t‐test, *P *< 0.01. *n *= 566). (B) The overexpression of *TOX* led to reduced expression of *TCF7*, *KLF2*, *LEF1*, and *CCR7*, and increased expression of *PDCD1*, *TIGIT*, *CTLA4*, *HAVCR2*, *TNFRSF9*, and *ID2*. (C) Line graph of apoptosis assays of tumor cells cocultured for 12 h using fluorescence‐activated cell sorting (*t*‐test, *P *< 0.01, *n *= 3) . Data are presented as means ± SD. (D) Proliferation efficiency of three types of CD8^+^ T cells: control (Ctrl), overexpressing (OE) CD8^+^ T cells, PDCD1 OE CD8^+^ T cells, and TOX‐overexpressing OE CD8^+^ T cells detected using carboxyfluorescein succinimidyl amino ester (CFSE).

Our results show that exhausted T cells were widespread in the OSCC microenvironment; therefore, to identify the key trans‐active regulators involved in the regulation of intratumoral T‐cell exhaustion, we focused on transcription factors among the differentially expressed genes (DEGs) as shown in Fig. 2C. Interestingly, TOX, which was recently reported to be a key regulator of CD8^+^ T cells in chronic infection [55, 56], was successfully retrieved among several TFs. This further highlighted the effectiveness of predicting key regulatory factors using scRNA‐seq data. Interestingly, among the ten genes that were strongly positively correlated with thymocyte selection‐associated high‐mobility group box (*TOX*) expression in our dataset, four genes (*CTLA4*, *TIGIT*, *TNFRSF9*, and *PDCD1*) were associated with T‐cell exhaustion (Fig. S7A), indicating that TOX is a key regulator of T‐cell exhaustion in OSCC. Moreover, the distribution patterns of TOX‐high cells and PDCD1‐high cells were similar in the t‐SNE plot (Fig. S2) and violin plot (Fig. 2C, upregulated in Clusters 4, 5, and 9), which were similar to the distribution patterns of other immune checkpoint genes such as *CTLA4* and *HAVCR2*. Furthermore, *TOX* and immune checkpoint genes (*PDCD1, CTLA4, HAVCR2, and LAG3*) exhibited similar expression dynamics along the developmental trajectories of CD8^+^ clusters in pseudotime analysis (Fig. 3B). More importantly, in the TCGA HCSN patient cohort, we observed a significant positive correlation between the expression of the above genes and that of *TOX* (t‐test, *P* <0.01, Figs 7A and S7B). However, naïve associated genes such as *KLF2, IL7R, CCR7, TCF7, and LEF1* were negatively correlated with the expression of *TOX*. As shown in Fig. 7B, consistent with our scRNA‐seq data, overexpression of *TOX* led to reduced expression of genes encoding naïve proteins (*TCF7, KLF2, LEF1, and CCR7*) and increased expression of genes encoding inhibitory receptors (*PDCD1, TIGIT, CTLA4, HAVCR2, and TNFRSF9*) and transcription factors such as ID2. As TOX expression was strongly correlated with the exhaustion of CD8^+^ T‐cell exhaustion, we explored the effect of TOX on the antitumor function of human T cells in vitro.

First, we cocultured PDL1^+^ tumor cells (HSC‐3 and CAL‐33) with Ctrl OE‐activated CD8^+^ T cells, PD1 OE‐activated CD8^+^ T cells, TOX OE‐activated CD8^+^ T cells, or activated human TOX OE CD8^+^ T cells with aPD1. Tumor cells were collected at 4, 8, and 12 h, and apoptosis assays were performed. The above cocultivation experiment was repeated 3 times. As shown in Fig. 7C (t‐test, *P* <0.01) and S7C, after 12 h of coculture, the apoptosis rate of tumor cells in the PD1 OE and TOX OE groups (6.41% and 7.87%, respectively, for HSC‐3, and 7.76% and 7.83%, respectively, for CAL‐33) was significantly lower than that in the Ctrl OE group and TOX OE plus anti‐PD1 group (21.29% and 23.02%, respectively, for HSC‐3, and 16.90% and 16.98%, respectively, for CAL‐33). The effect of TOX overexpression on CD8^+^ T cells could be reversed by aPD1, indicating the significant role of the PD1/PDL1 axis in the reduced cytotoxic activity due to TOX overexpression.

We also cultured three types of human CD8^+^ T cells (Ctrl OE CD8^+^ T cells, PD1 OE CD8^+^ T cells, and TOX OE CD8^+^ T cells) in T‐cell culture medium under CD3/CD28 stimulation. T cells were labeled with CFSE, collected on day 2, and their proliferation was measured using a flow cytometer. As shown in Fig. 7D, PD1 OE CD8^+^ T cells and TOX OE CD8^+^ T cells had diminished proliferation (20.4% and 18.2%, respectively) compared with that of Ctrl OE CD8^+^ T cells (78.2%).

The above findings revealed that the cytotoxic activity and proliferation efficiency of CD8^+^ T cells overexpressing TOX or PD1 were reduced, consistent with studies by Wing *et al*. [29], Parry *et al*. [30], and Buchbinder and Desai [57]. In addition, considering the TCGA expression profile (Fig. 7A,B) and in vitro experimental results (Fig. 7B–D) combined with related reports on the effect of TOX on dysfunction‐associated gene expression [55, 58], we conclude that TOX is a critical regulator of T‐cell differentiation and is positively correlated with the expression of dysfunction‐associated genes.

### 3.8 Transcription factor network analysis revealed PRDM1 as a key regulon for TOX

**Fig. 8:**
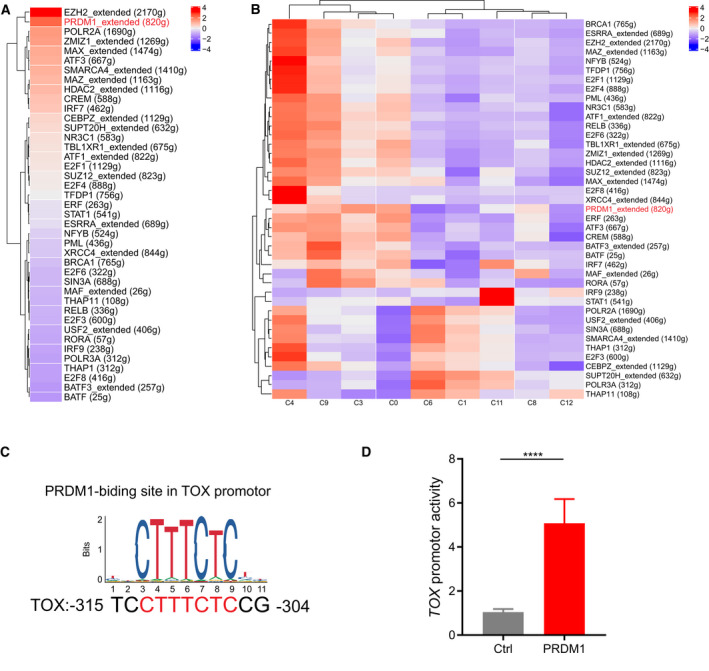
PRDM1 activated TOX gene transcription. (A) Transcription factor networks that drive the expression of *TOX* in total CD8^+^ T cells predicted by SCENIC. (B) Heatmap of regulators predicted by SCENIC that drives the expression of *TOX* in CD8^+^ T cells clusters. The PRDM1 regulon is indicated in red. (C) A conserved PRDM1‐binding motif at the TOX promoter was predicted by JASPAR. (D) Dual‐luciferase reporter assays of Jurkat cells transfected with PRDM1 overexpression plasmid and reporter plasmid containing TOX promoter (ANOVA, *P *< 0.0001, *n *= 3). Jurkat cells transfected with a blank pGL3 plasmid served as a negative control. Data are presented as means ± SD.

Since TOX was shown to have a key role in T‐cell dysfunction, we used single‐cell regulatory network inference and clustering (SCENIC) to study the underlying molecular mechanisms that drive the expression of TOX. For total CD8^+^ T cells, the PRDM1 regulon was among the top three regulators that SCENIC predicted to drive TOX expression (Fig. 8A). Moreover, CD8^+^ T‐cell clusters upregulated the expression of different transcription factor networks that drive the TOX expression, with PRDM1 regulators showing high specificity in cluster 3, which express high levels of genes associated with dysfunction (Figs 8B and S8). This suggests that TF PRDM1, which had been identified as TFs with function related to T‐cell exhaustion [59], but had not been reported in T cells infiltrating in OSCC, may be responsible for the transcription of TOX. To this end, we used the JASPAR database to reveal a canonical binding motif for transcription factor PRDM1 in the TOX promoter (Fig. 8C). To confirm this, we electronically transfected Jurkat cells, a human lymphocyte cell line with PRDM1 overexpression plasmid and pGL3 reporter plasmids containing the *TOX* promoter. A dual‐luciferase reporter assay showed that PRDM1 activated *TOX* gene transcription (ANOVA, *P*<0.0001, Fig. 8D). The above findings revealed that PRDM1 is a key regulator of TOX expression in T cells infiltrating OSCC.

## 4 Discussion

Our transcriptome data for >10,000 individual T cells constitute a comprehensive resource for understanding the multidimensional characterization of T cells and significantly contribute to understanding the immune infiltration landscape of the TME. The higher resolution provided by our dataset can be exemplified by the identification of 14 large subsets and unique subpopulations, such as exhausted CD8^+^ T cells, and the high quantity and quality of single‐cell data that allowed us to map their developmental trajectory.

Previous studies have mentioned that the preferential accumulation of both CD4^+^ Tregs and exhausted CD8^+^ T cells in OSCC may be a result of local expansion of these cells [60, 61]. We showed, based on the results of marker genes, pseudotime, and GO analysis, that although CD8^+^ T cells in adjacent normal tissue expressed functions of ‘cell activation’, ‘inflammatory effect’, and ‘immunity effect’ (Figs 5C and S6B), they did not express functions such as ‘cell cycle’ and ‘cell proliferation’. In contrast, the ‘no‐exhausted’ CD8^+^ T cells in the tumor actively expressed ‘cell cycle’ and ‘cell proliferation’ functions (Figs 5A and S5). Similar results were obtained by comparing CD4^+^ T cells in the adjacent normal and tumor tissues (Figs 5B,D, S6A, and S6C). Our results suggest that exhausted CD8^+^ T cells and CD4^+^ Tregs in tumors likely evolved from other types of CD8^+^ and CD4^+^ T cells inside the tumor.

T‐cell infiltration and characteristics are usually associated with different prognostic outcomes, and T cells in the TME will evolve in different directions when exposed to different antigen receptor signaling and cytokine stimulation [62]. Using pseudotime analysis, we found that the ‘initially’ nonfunctional T cells in tumor tissues evolved in different directions after receiving the ‘T cell activation’ signal. For example, cluster 4_CD8^+^ mitotic T_EM_ and cluster 7_CD4^+^ T_EM_ exerted effective immune functions, whereas cluster 3_CD8^+^ T exhausted and cluster 2_CD4^+^ Treg were immunosuppressed after the inhibitory program was activated. In addition, the specific expression of TOX in cluster 4_CD8^+^ mitotic T_EM_ and cluster 9_CD4^+^ T_EM_ suggests that TOX is specifically required for T‐cell differentiation in the TME of OSCC and that this affects the expression of other inhibitory receptors on T cells such as that of PD1. As Seo *et al*. and Scott *et al*. suggested, interfering with the expression or function of TOX may prove to be an important therapeutic strategy for cancer immunotherapy in the future [58, 63]. Combining the results of IHC, clustering, pseudotime, GO analysis, and in vitro experiments, we conclude that the evolution of T cells toward immunosuppression is mediated by TOX, PDCD1, CTLA4, and CD28, suggesting that T cells could be prevented from becoming immunosuppressive by ‘blocking’ these molecules. As Sharma and Allison suggested, unleashing the power of T cells is important for cancer immunotherapy [62].

Cluster 12_CD8^+^ T_EM_ appears to be different from conventional T‐cell subtypes. Compared with effector T cells, the cells of cluster 7_CD8^+^ T_EM_ expressed lower levels of cytotoxic marker genes (*GNLY*, *IFNG*, and *TNFRSF18*) and certain levels of exhaustion marker genes (*CTLA4* and *STAT2*), which could indicate that the cells of cluster 12_CD8^+^ T_EM_ are in a transitional state from effector to exhausted T cells. Therefore, promoting the transition of these cells to effector‐like cells and preventing them from exhaustion might be a possible therapeutic strategy. Notably, an antibody blockade of the PD1 pathway has been shown to reinvigorate exhausted CD8^+^ T cells with intermediate expression of PD1, whereas those with high PD‐1 expression are unable to recover [64]. In addition to cluster 12_CD8^+^ T_EM_, we identified cluster 5_CD4^+^ T_EM_ cells that shared gene expression characteristics similar to those of CD8^+^ effector T cells, implicating their cytotoxic potential. These cells will evolve into Tregs and effector T cells as the immune state develops (Fig. 3B). Therefore, promoting these cells to resemble effector T cells and preventing them from becoming exhausted T cells [65] might be another strategy for OSCC immunotherapy.

The immunomodulatory drug, anti‐PD1, which was approved in 2016 by the United States Federal Drug Agency for treating HNSCC, was approved for the same use in China in 2019. Based on the results of previous studies [66, 67, 68] and our study, we conclude that the TME of OSCC is highly complex with a dramatically altered gene expression pattern of exhausted T cells, and therefore, the effect of a single‐agent anti‐PD1 therapy may not be ideal. Thus, exploring the upstream regulatory mechanism of T‐cell exhaustion and the distinctive features of the TME may provide a foundation for the design of therapies targeting OSCC. PRDM1 and TOX, which have rarely been studied in OSCC to date, have been highlighted as prominent upstream regulatory mechanisms in T‐cell exhaustion in OSCC. This suggests that immunotherapy needs to simultaneously attack the tumor from multiple angles while avoiding malignant changes in the adjacent normal tissue.

## 5 Conclusion

In summary, to the best of our knowledge, this work represents a unique resource providing a comprehensive single‐cell transcriptome atlas of the T cell‐infiltrated TME in OSCC. This study lays a new foundation for the development of precision immunotherapies for OSCC.

## 6 Ethics declarations

### Ethics approval and consent to participate

The study was reviewed and approved by the Medical Ethics Committee of Sun Yat‐Sen University. Since all the specimens used were anonymous, the Medical Ethics Committee exempted patients from the need for informed consent.

## Conflict of interest

The authors declare no conflict of interest.

## Author contributions

JTC, SWC, and MS designed the study. JTC, JFY, and HL planned the experiments. JTC, JFY, XYL, and JW carried out the experiments. MS, XZ, and YZ contributed to surgical excision of tissue samples and histopathological confirmation. JTC, JFY, HL, SWC, and MS contributed to the interpretation of the results. JTC, JFY, ZYY, and JW validated the results. JTC and JFY wrote the original manuscript. HL, SWC, and MS reviewed, edited, and finalized the manuscript. All authors provided critical feedback and helped shape the research, analysis, and manuscript.

### Peer Review

The peer review history for this article is available at https://publons.com/publon/10.1002/1878‐0261.12910.

## Supporting information


**Fig S1.** Isolation of live tumor infiltrating lymphocytes using fluorescence‐activated cell sorting.

**Fig S2.** Feature plots and gene heat map of T cell sub‐clusters.

**Fig S3.** The pseudotime state related to Figure 3.

**Fig S4.** Gene Ontology (GO) Biological process bar‐plot related to immune pathways of tumor tissue cluster 0 and 2.

**Fig S5.** Feature plots of key genes in normal tissue T cell clusters related to Figure 5A.

**Fig S6.** Supplemental analysis for normal tissues.

**Fig S7.** Genes related to *TOX* expression and the effect of *TOX* on apoptosis.

**Fig S8.** Regular specificity scores for relevant genes driving *TOX* expression.

**Fig S9.** Overexpression of marker genes.

**Fig S10.** Quantification of Foxp3^+^ T cells (A) Representative flow cytometry plots showing the percentage of Foxp3^+^ T cells in control‐overexpressing human CD4^+^ T cells. Human CD4^+^ T cells were stimulated in vitro with Human T‐Activator CD3/CD28 Dynabeads for 36h and then infected with control‐overexprssing lenti‐virus. Tregs were induced by culturing the infected T cells in the presence of recombinant human TGF‐β plus IL‐2 for 4 days. (B) Representative flow cytometry plots showing the percentage of Foxp3^+^ T cells in TOX‐overexpressing human CD4^+^ T cells.

**Table S1.** (A‐B) List of The Cancer Genome Atlas (TCGA) cohorts of patients with head and neck squamous cell carcinoma (HNSCC) related to Figure 4C and Figure 4F.

**Table S2.** List of clinicopathological features of patients’ tissue samples used in immunohistochemistry.

## Data Availability

The authors confirm that all relevant data are included in the article and that materials are available upon request from the authors.
