# Supplementary figures and images for: Single‐cell transcriptomics reveal the intratumoral landscape of infiltrated T‐cell subpopulations in oral squamous cell carcinoma
Source: Mol Oncol. 2021 Mar 9;15(4):866–86. doi: 10.1002/1878-0261.12910 (PMC8024729; doi:10.1002/1878-0261.12910)

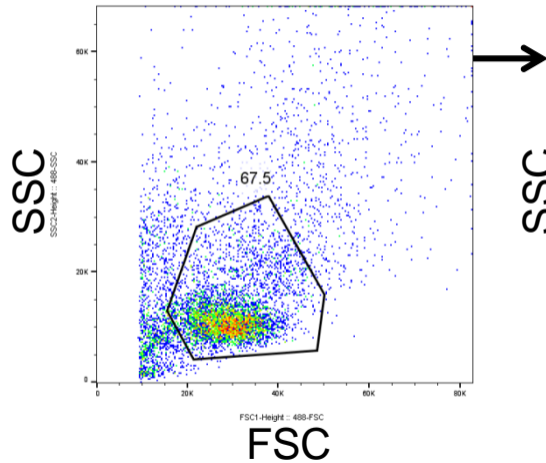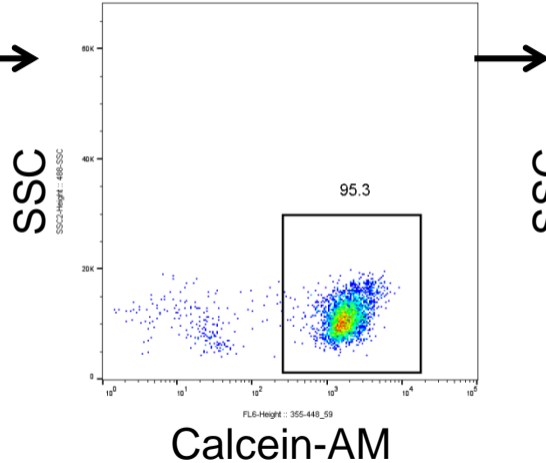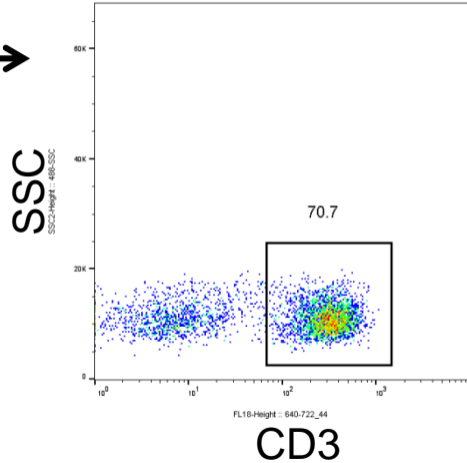

Supplement: Supplementary file 1 — Fig S1. Isolation of live tumor infiltrating lymphocytes using fluorescence‐activated cell sorting. [file MOL2-15-866-s002.pdf]

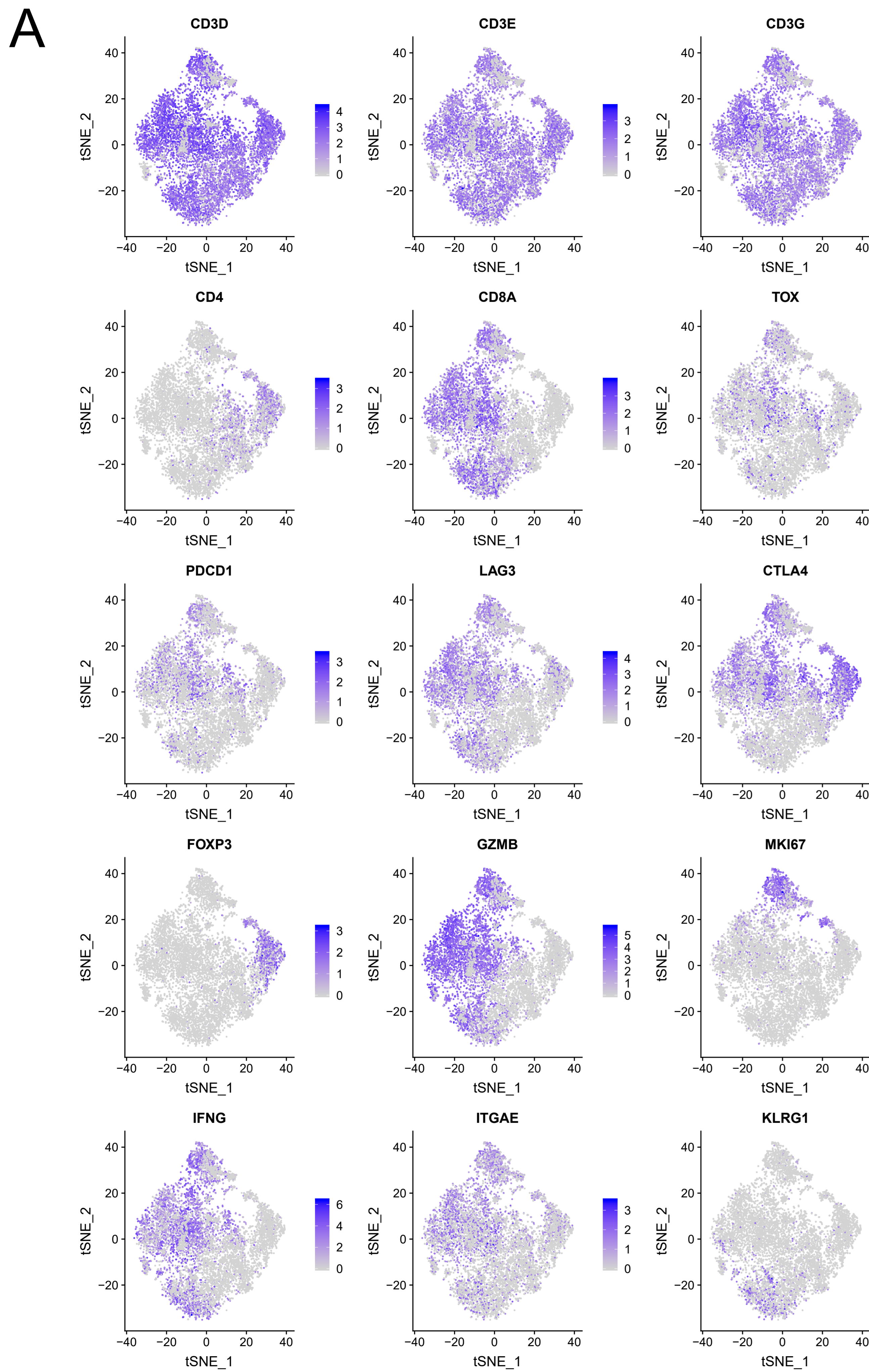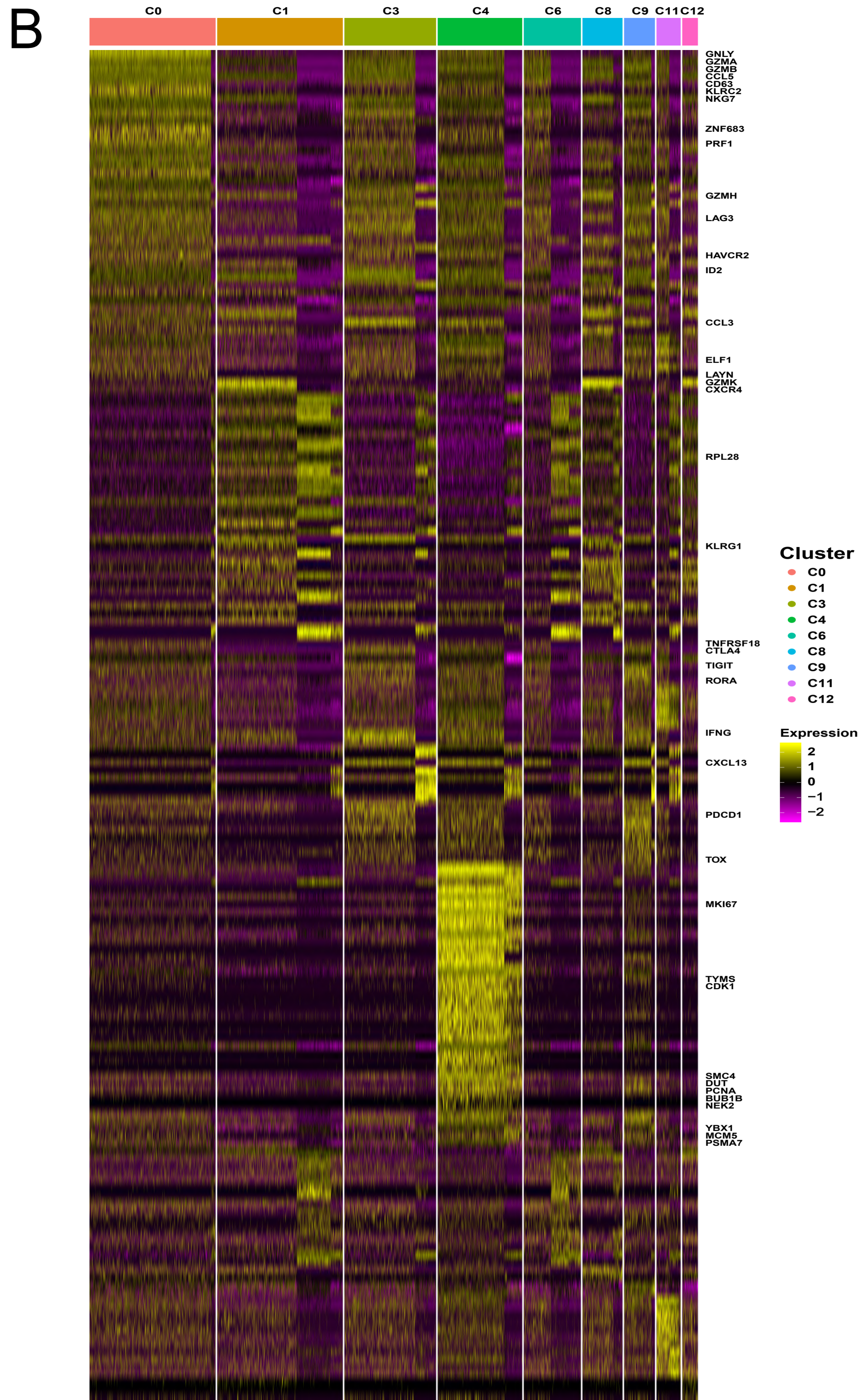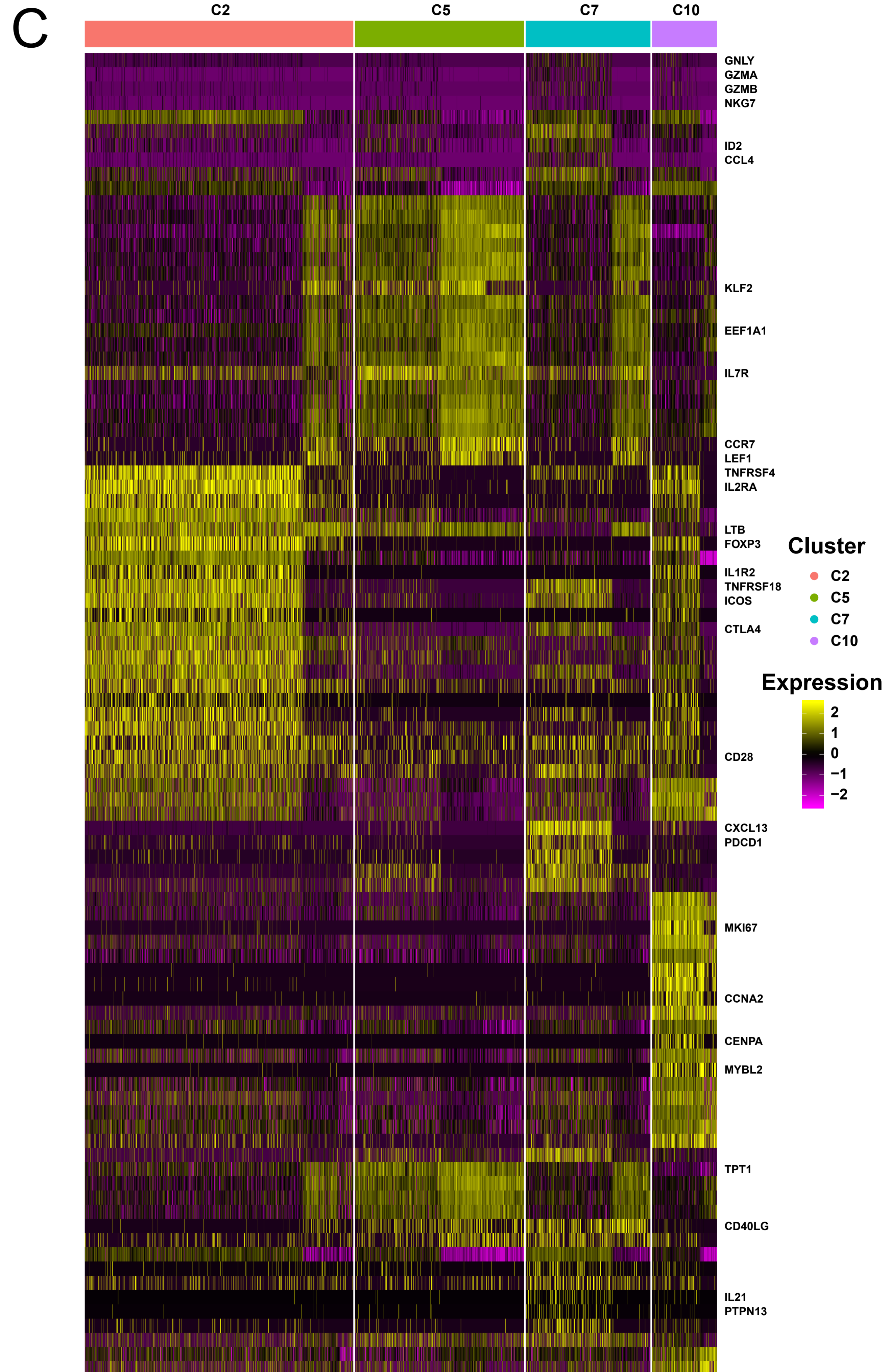

Supplement: Supplementary file 2 — Fig S2. Feature plots and gene heat map of T cell sub‐clusters. [file MOL2-15-866-s004.pdf]

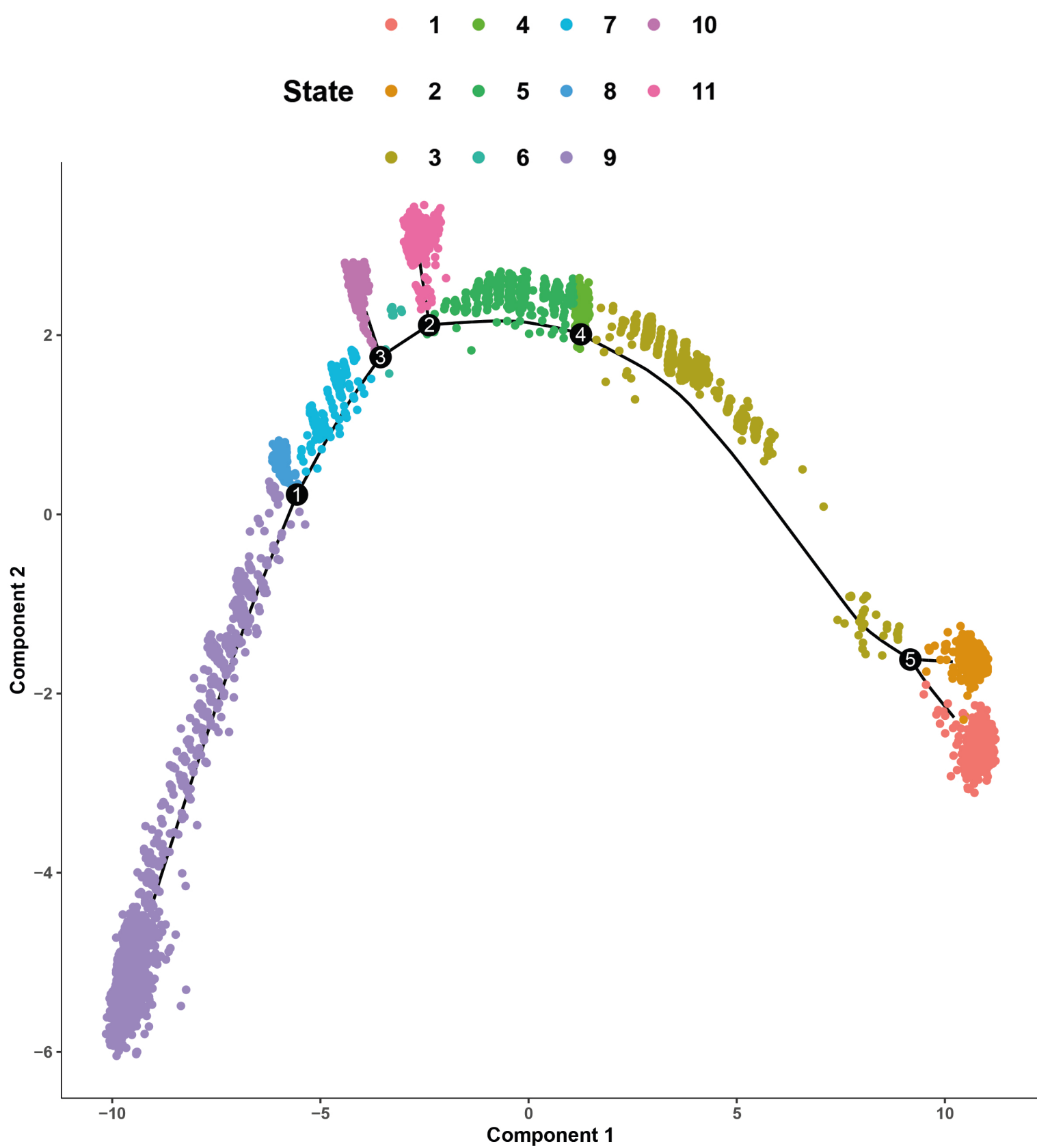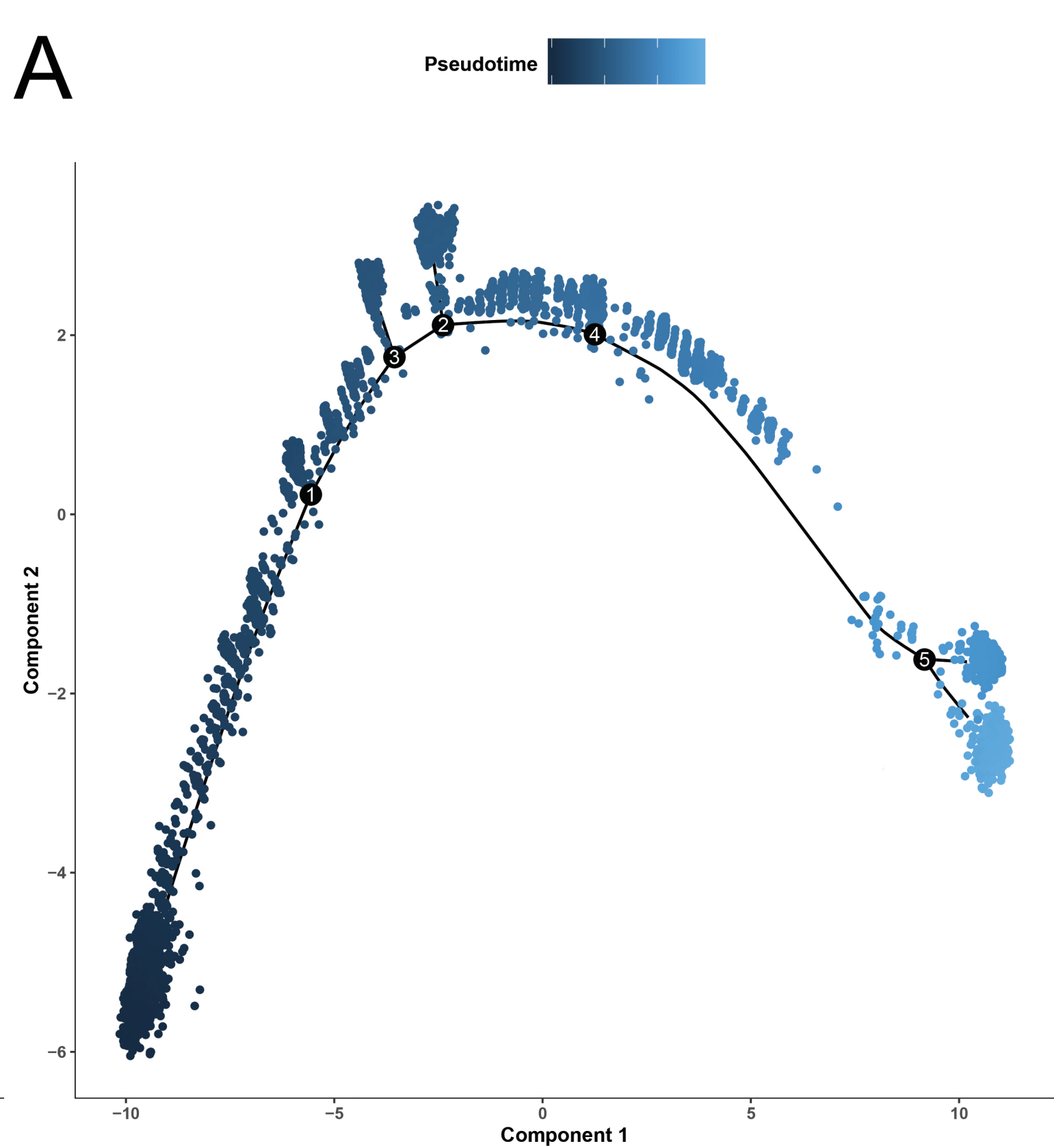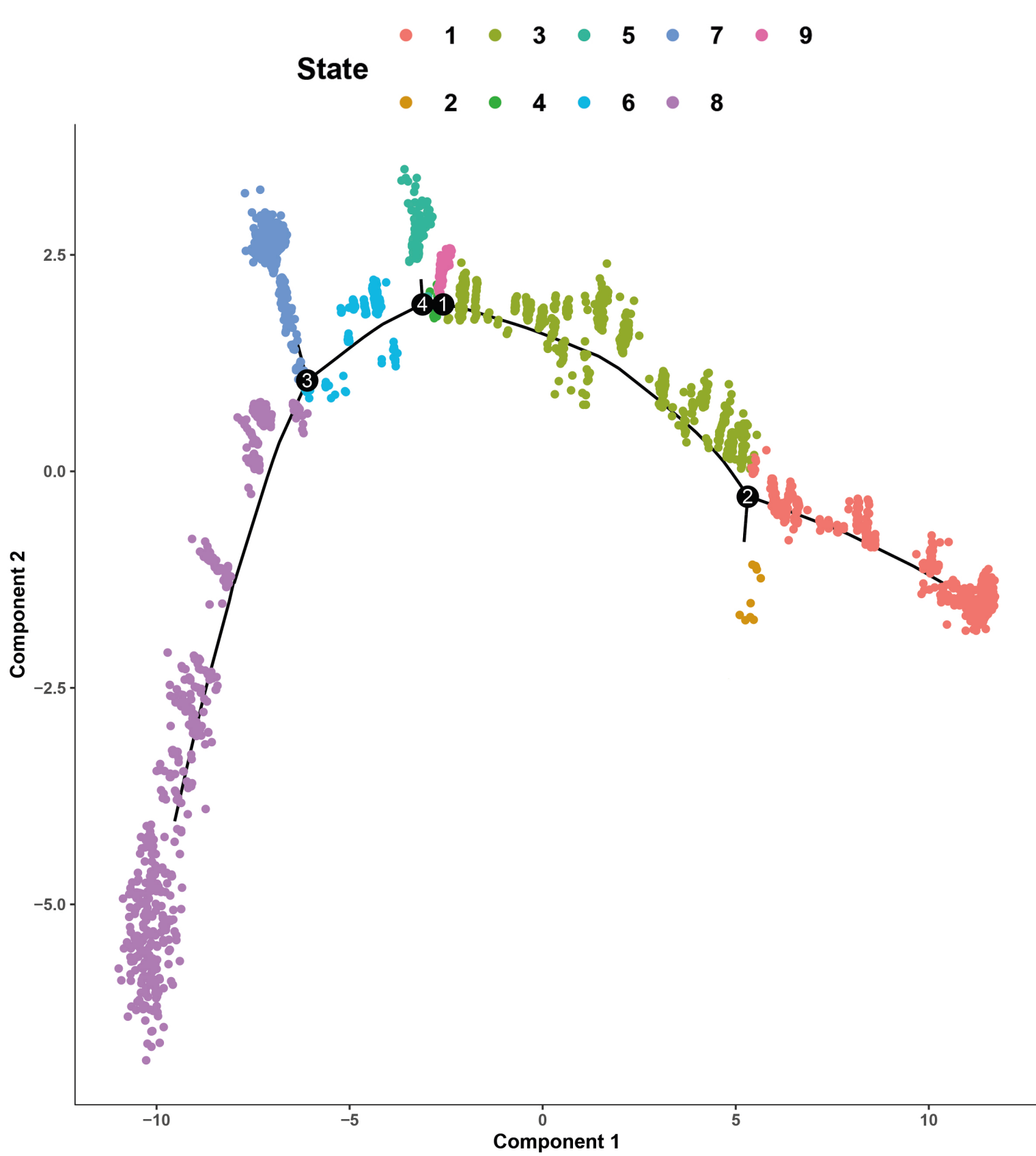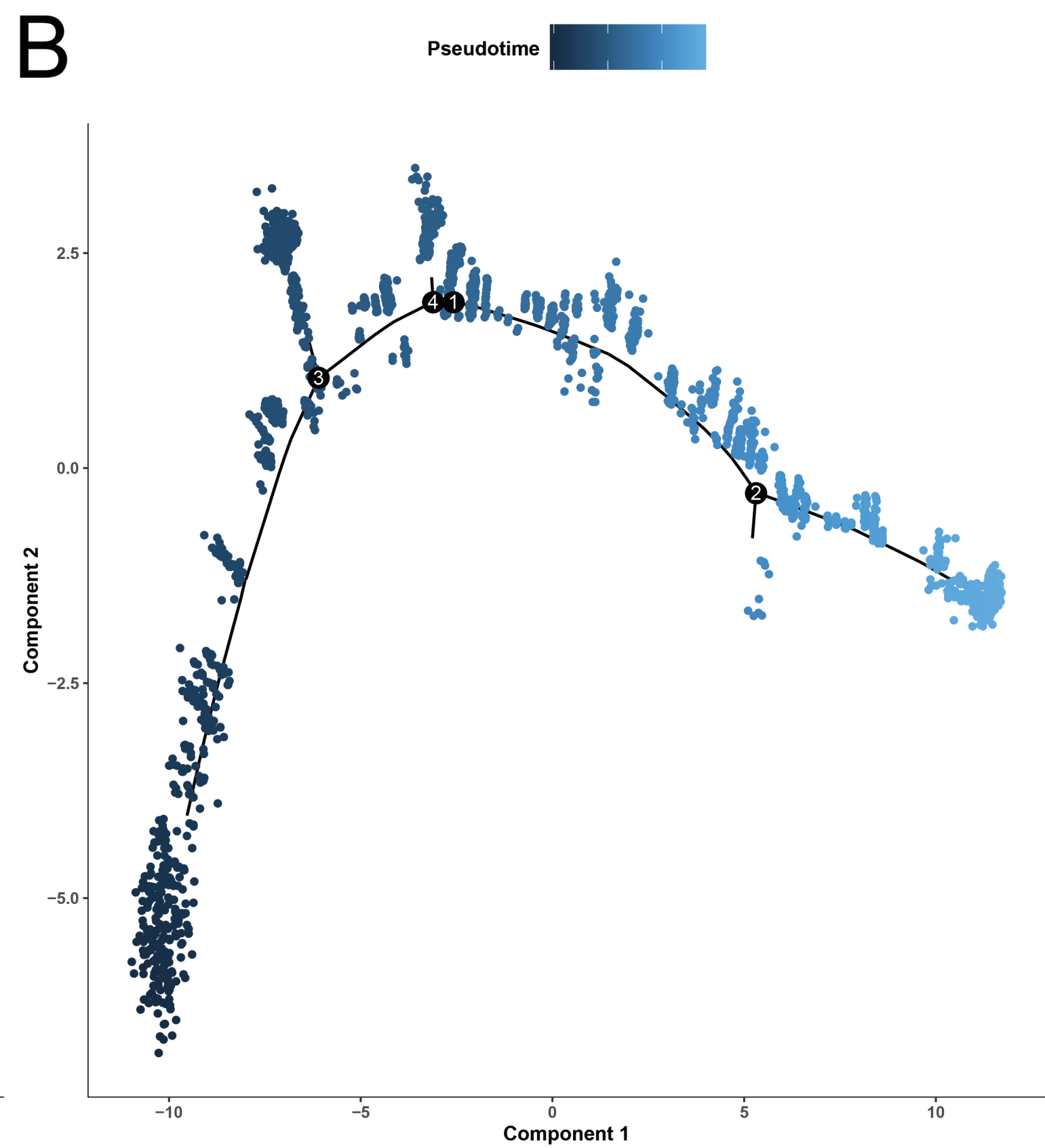

Supplement: Supplementary file 3 — Fig S3. The pseudotime state related to Figure 3. [file MOL2-15-866-s005.pdf]

A

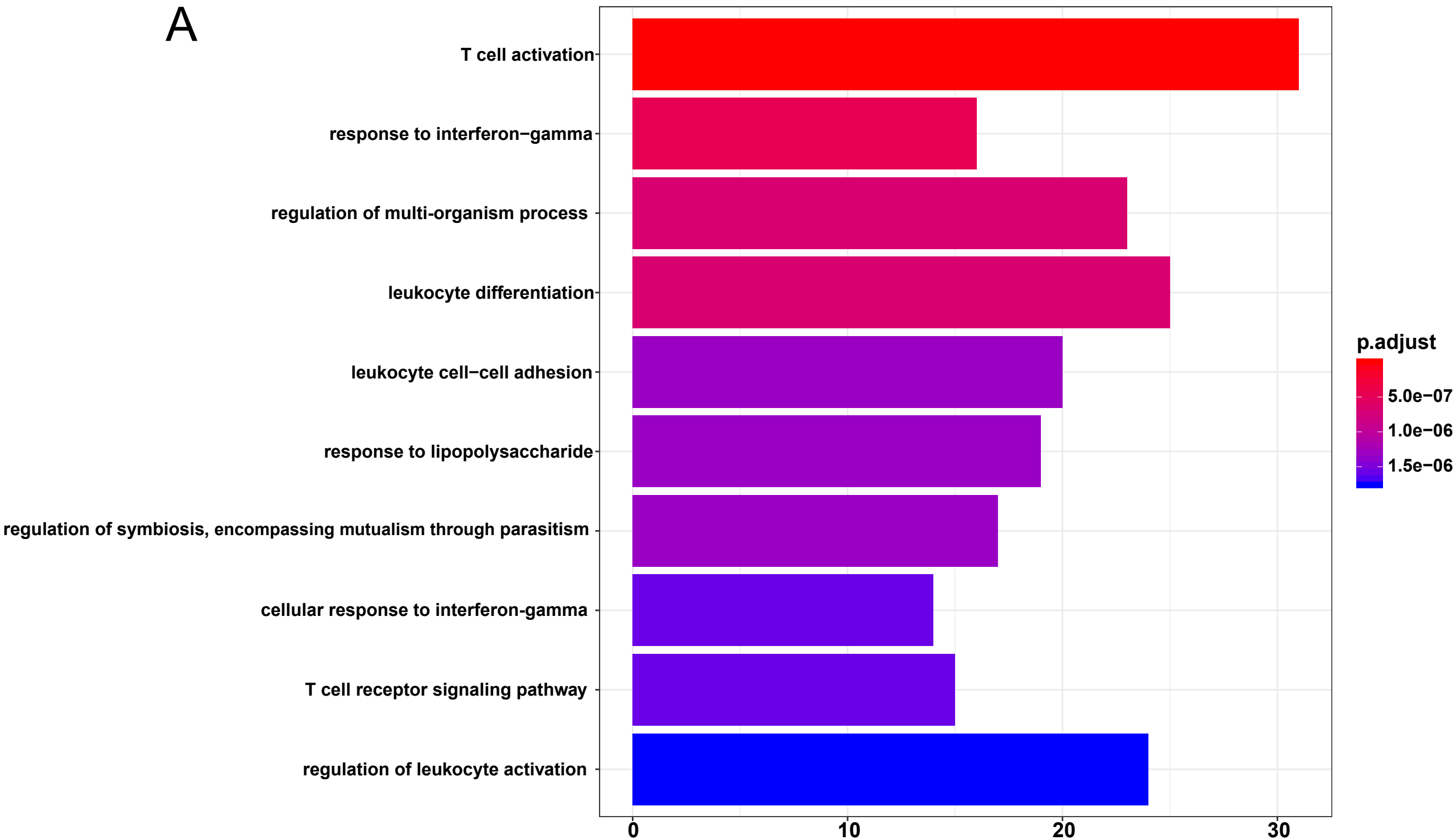

B

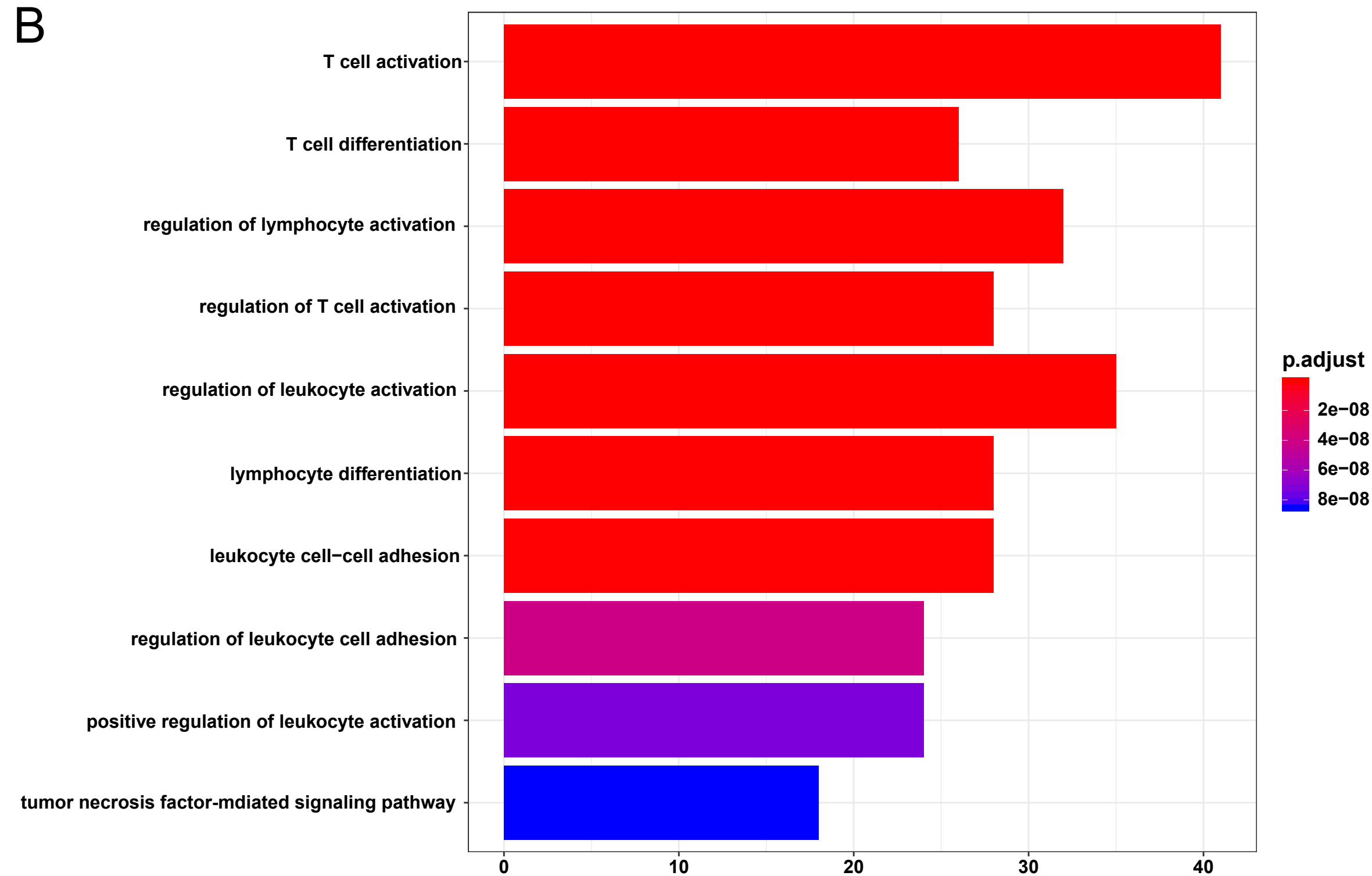

Supplement: Supplementary file 4 — Fig S4. Gene Ontology (GO) Biological process bar‐plot related to immune pathways of tumor tissue cluster 0 and 2. [file MOL2-15-866-s007.pdf]

CD3E

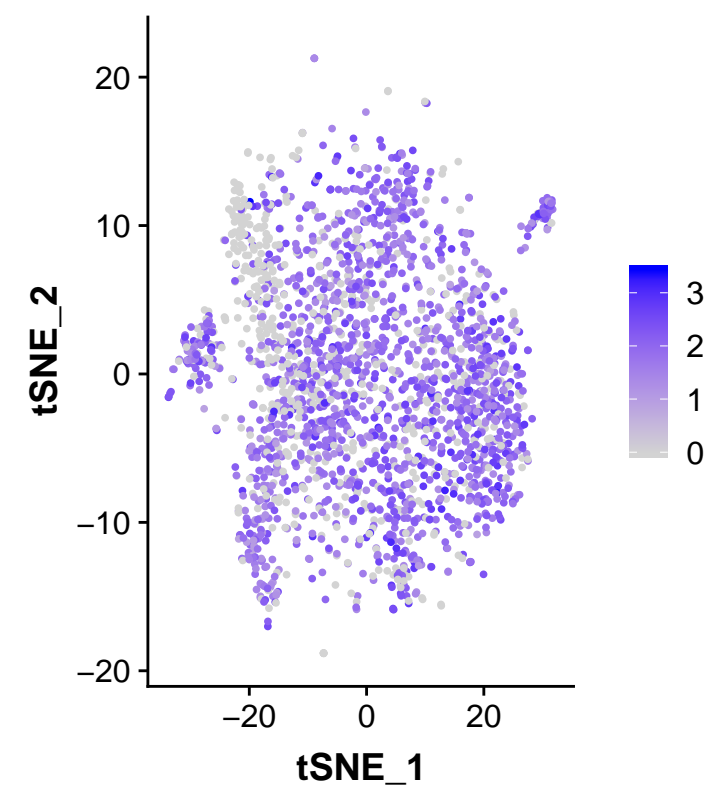

CD8A

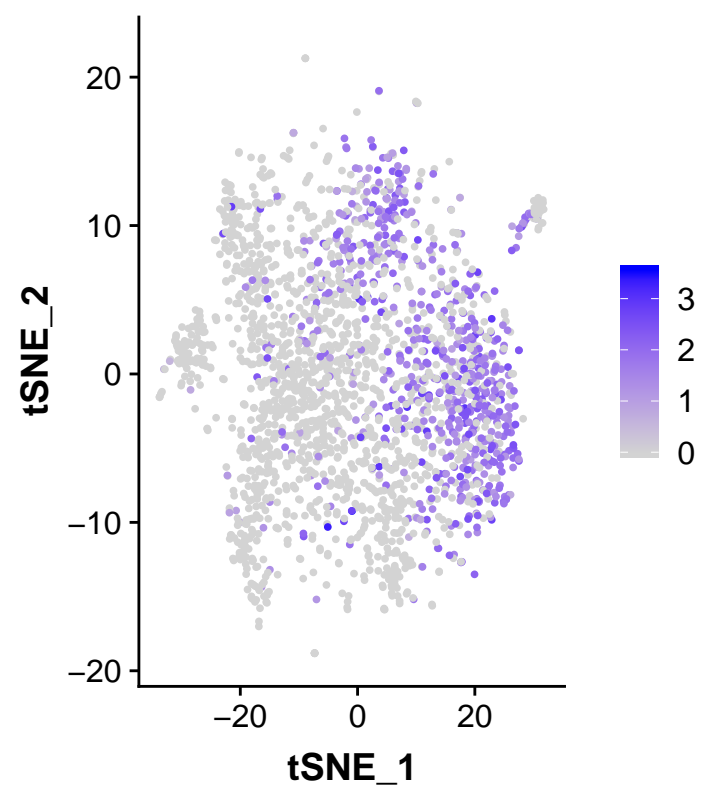

CD4

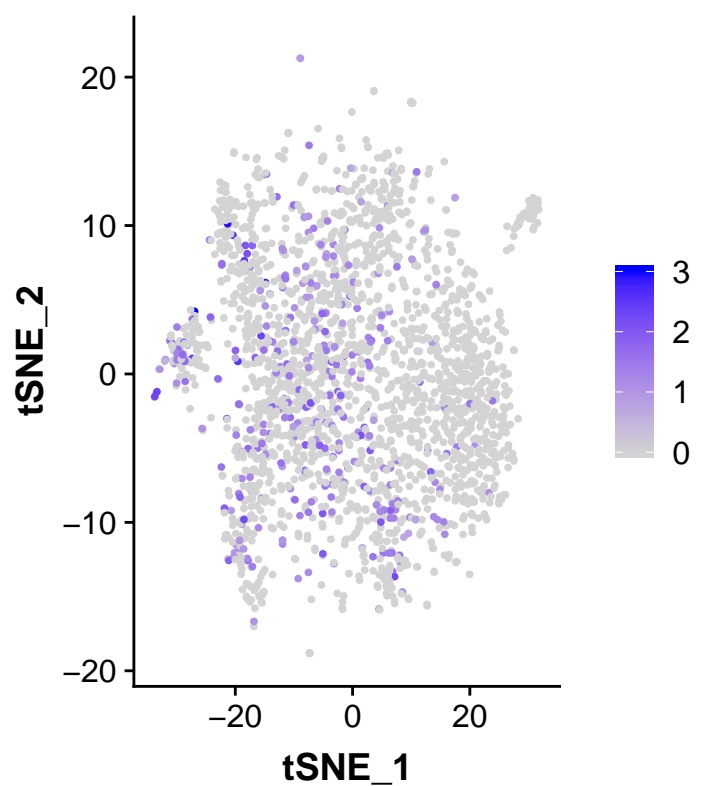

CD14

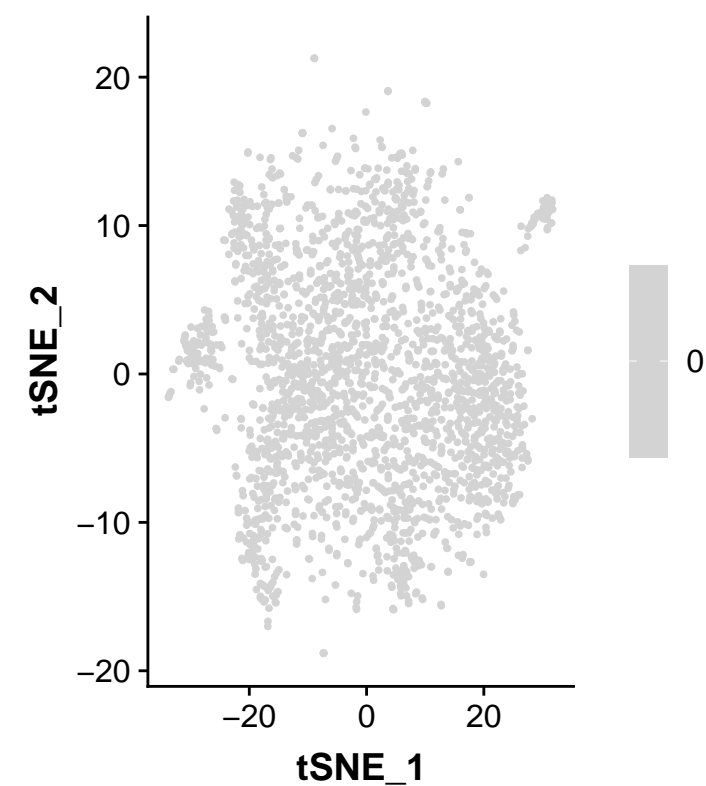

FOXP3

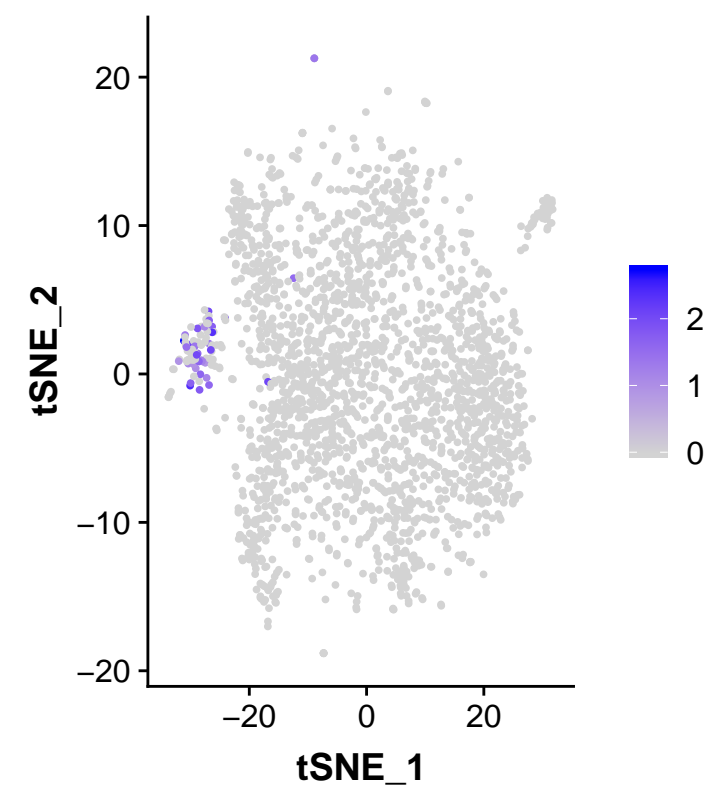

LAG3

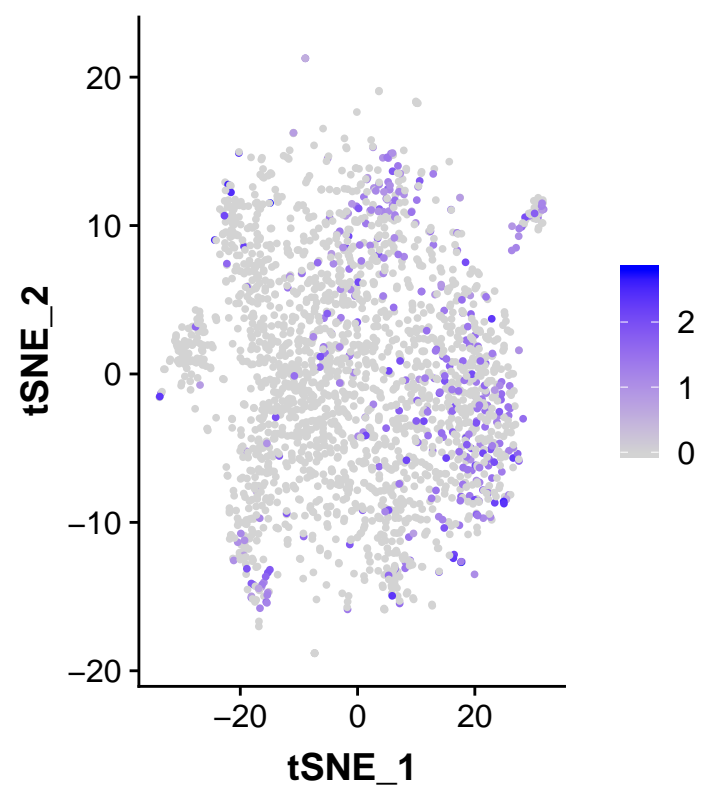

PDCD1

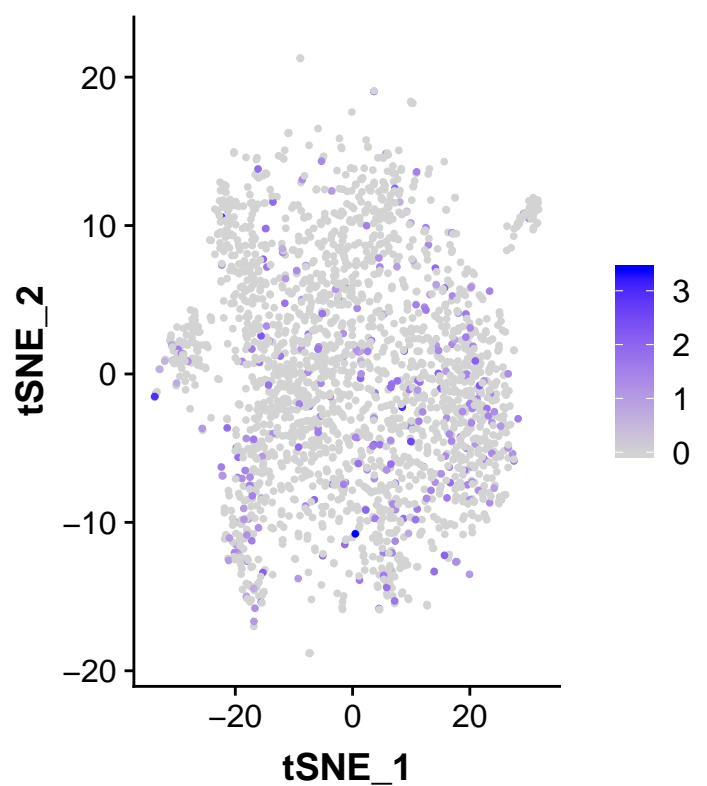

CTLA4

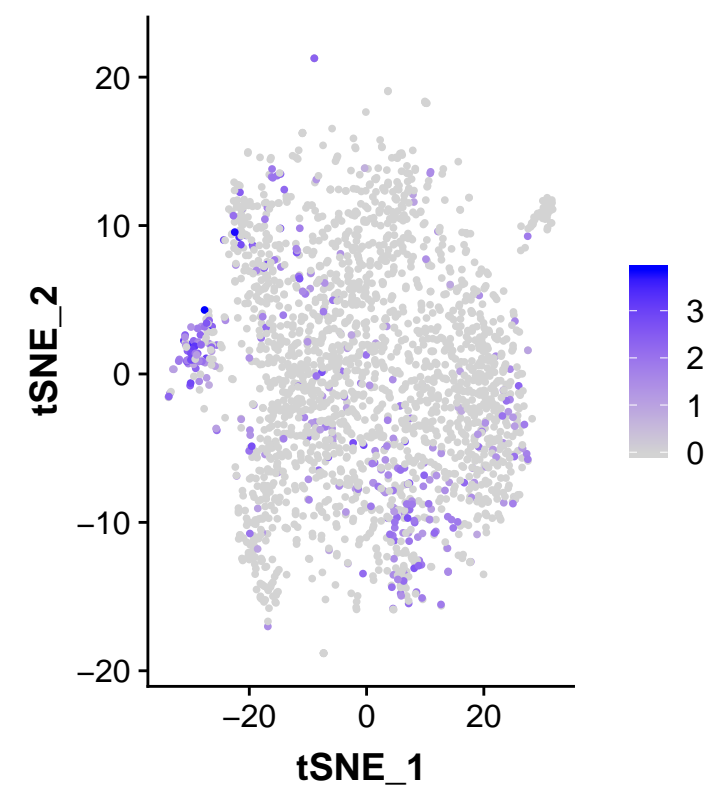

MKI67

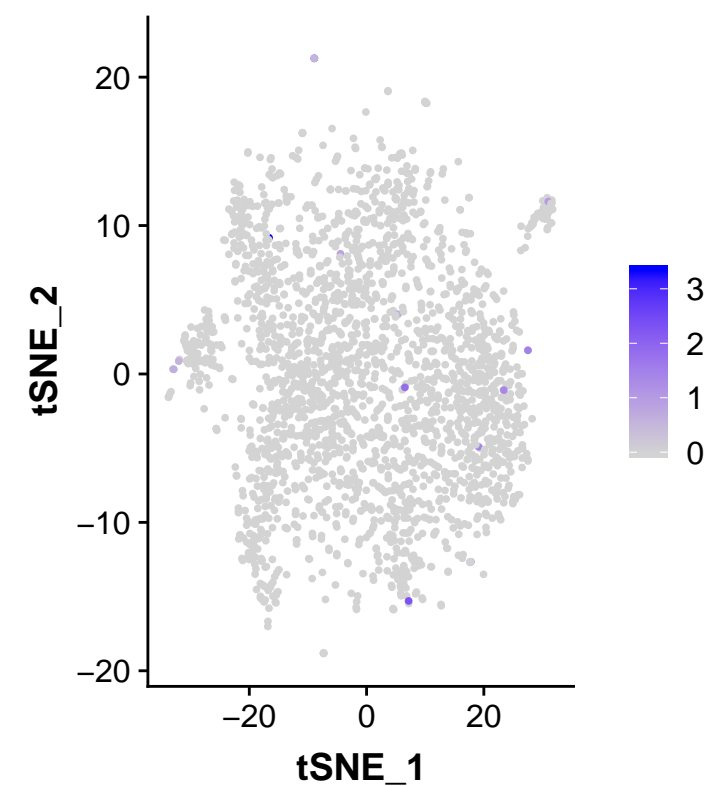

GZMA

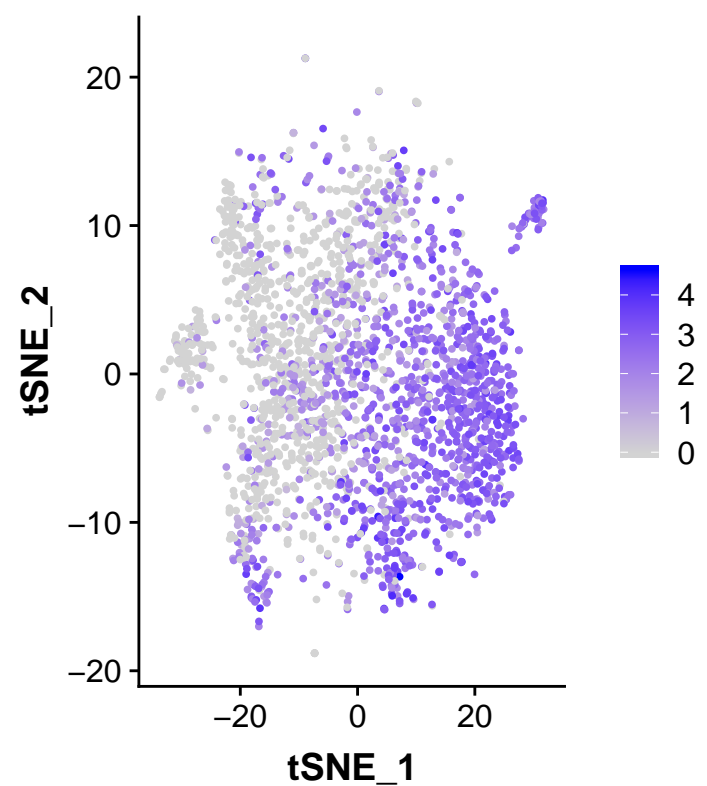

GZMB

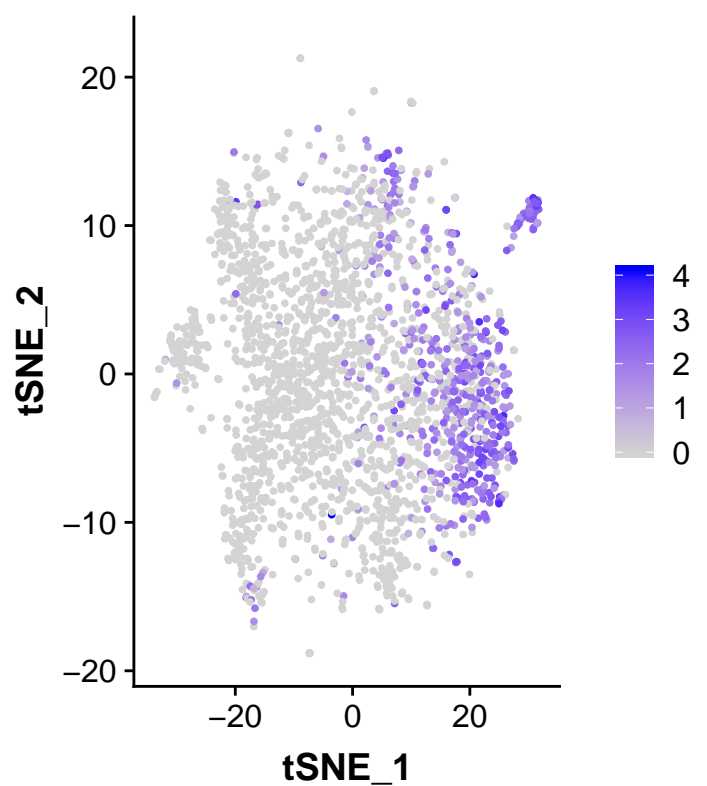

HMGB2

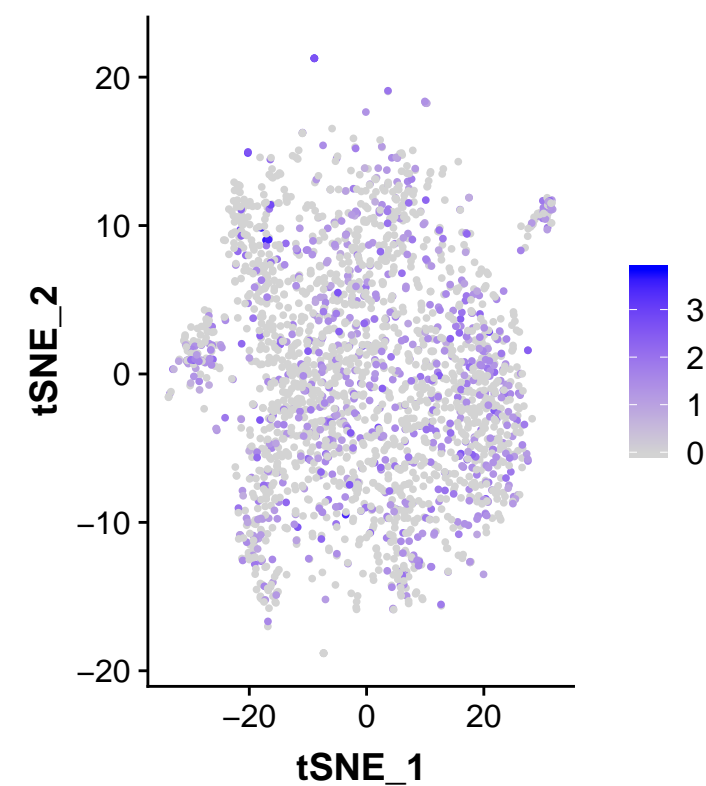

STMN1

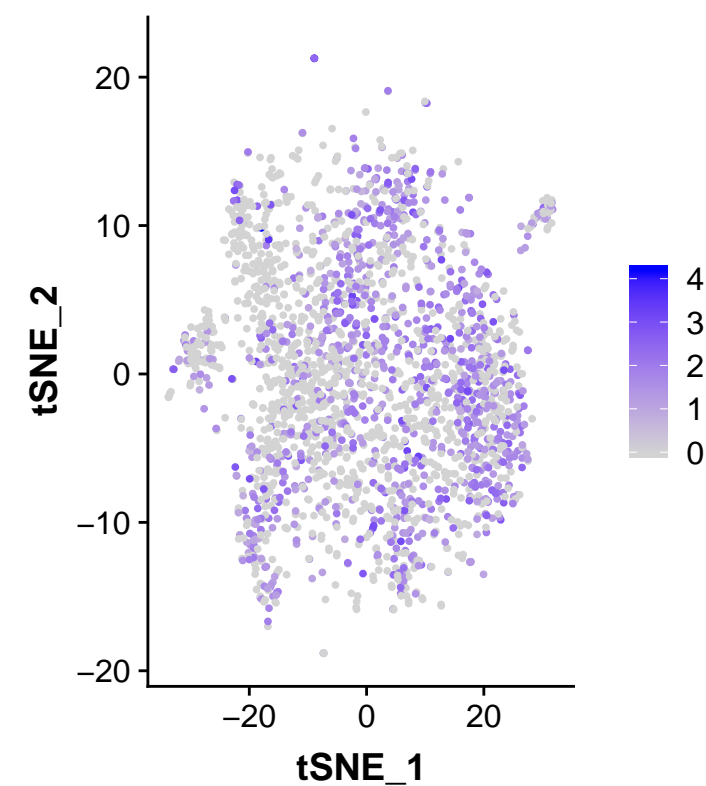

MKI67

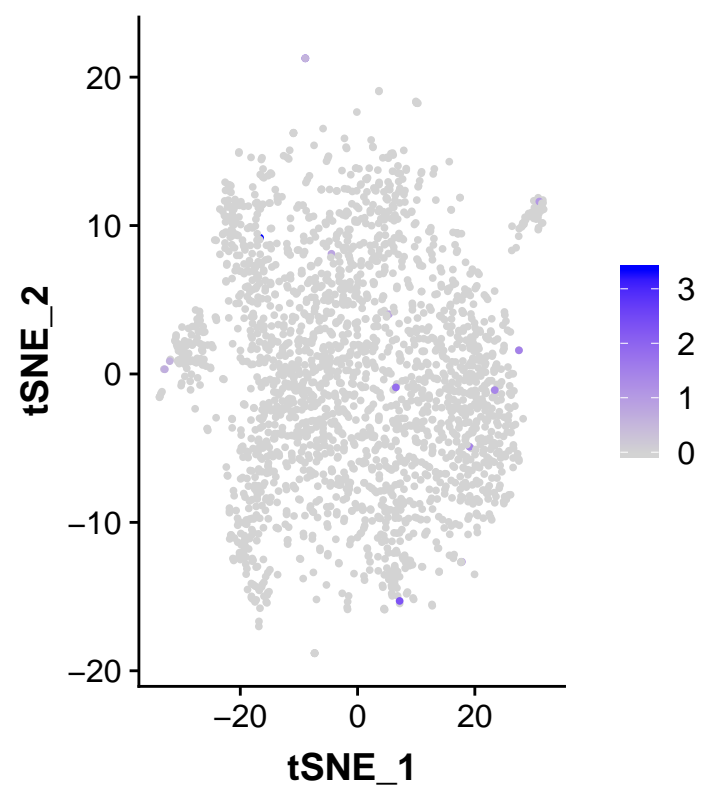

GINS2

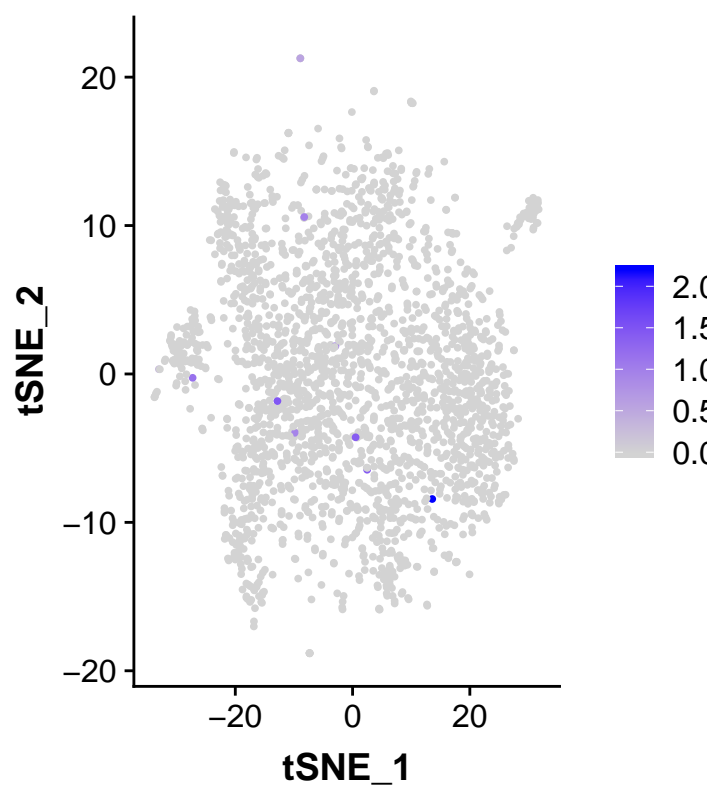

Supplement: Supplementary file 5 — Fig S5. Feature plots of key genes in normal tissue T cell clusters related to Figure 5A. [file MOL2-15-866-s006.pdf]

A

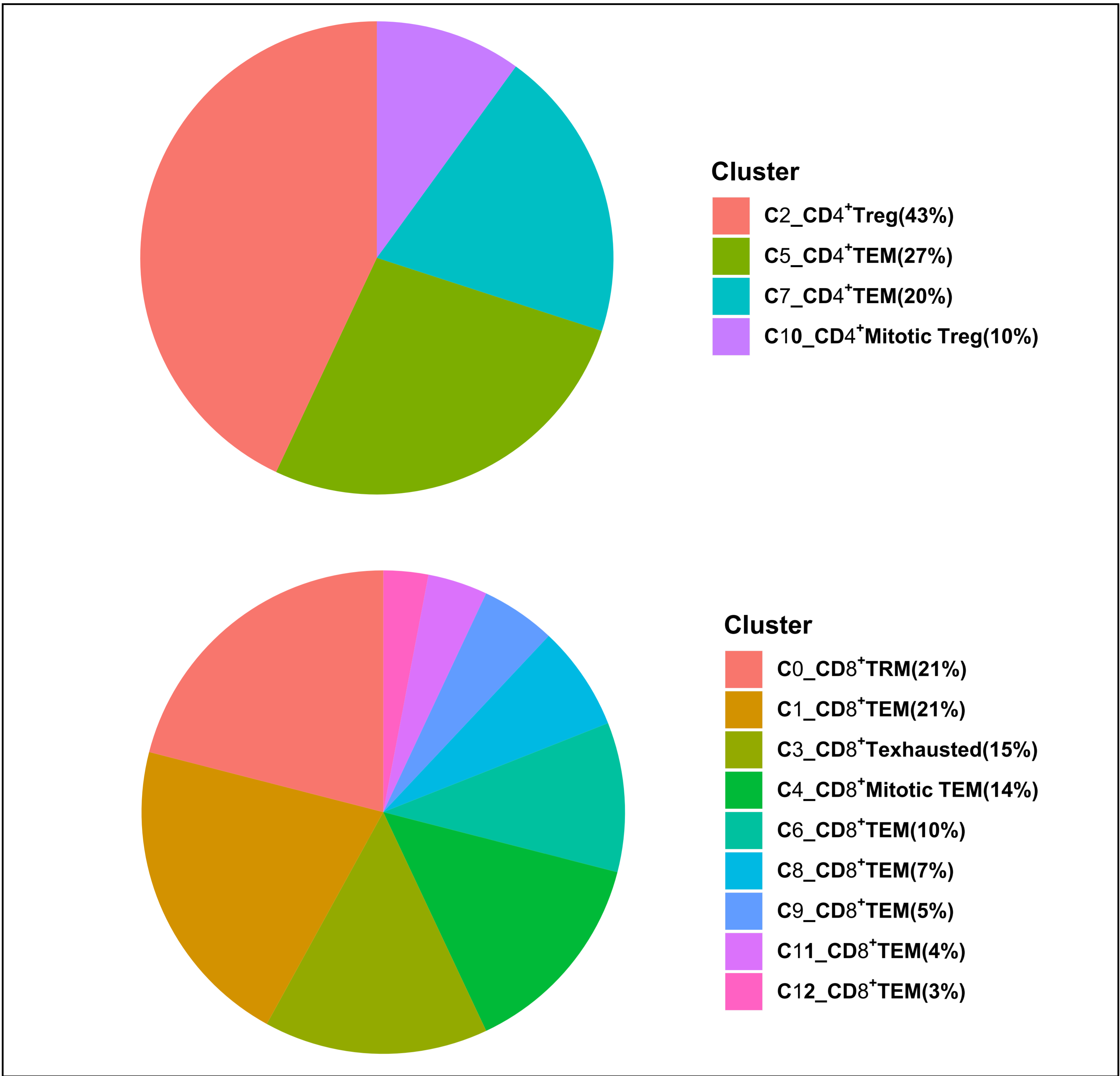

B

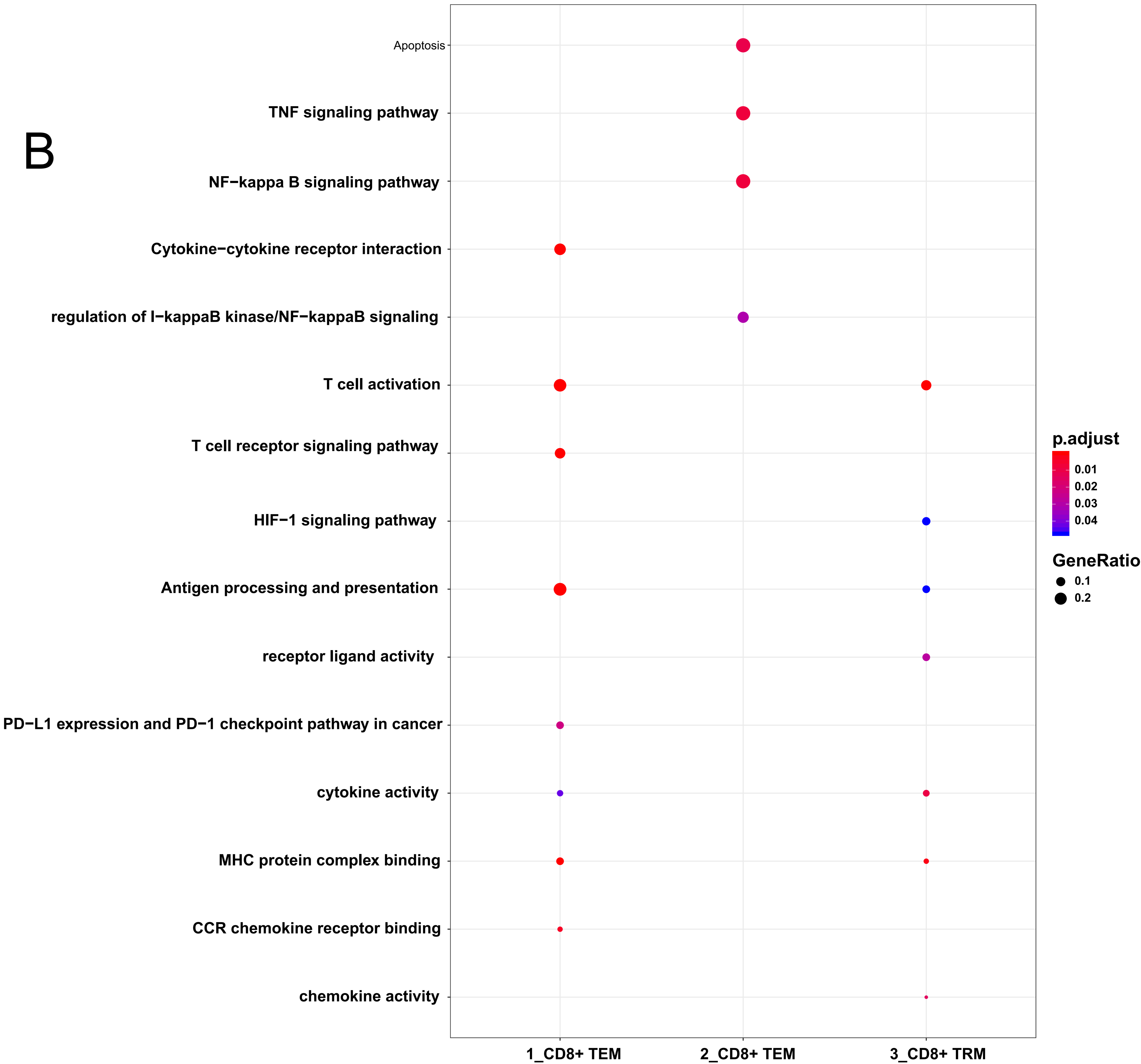

C

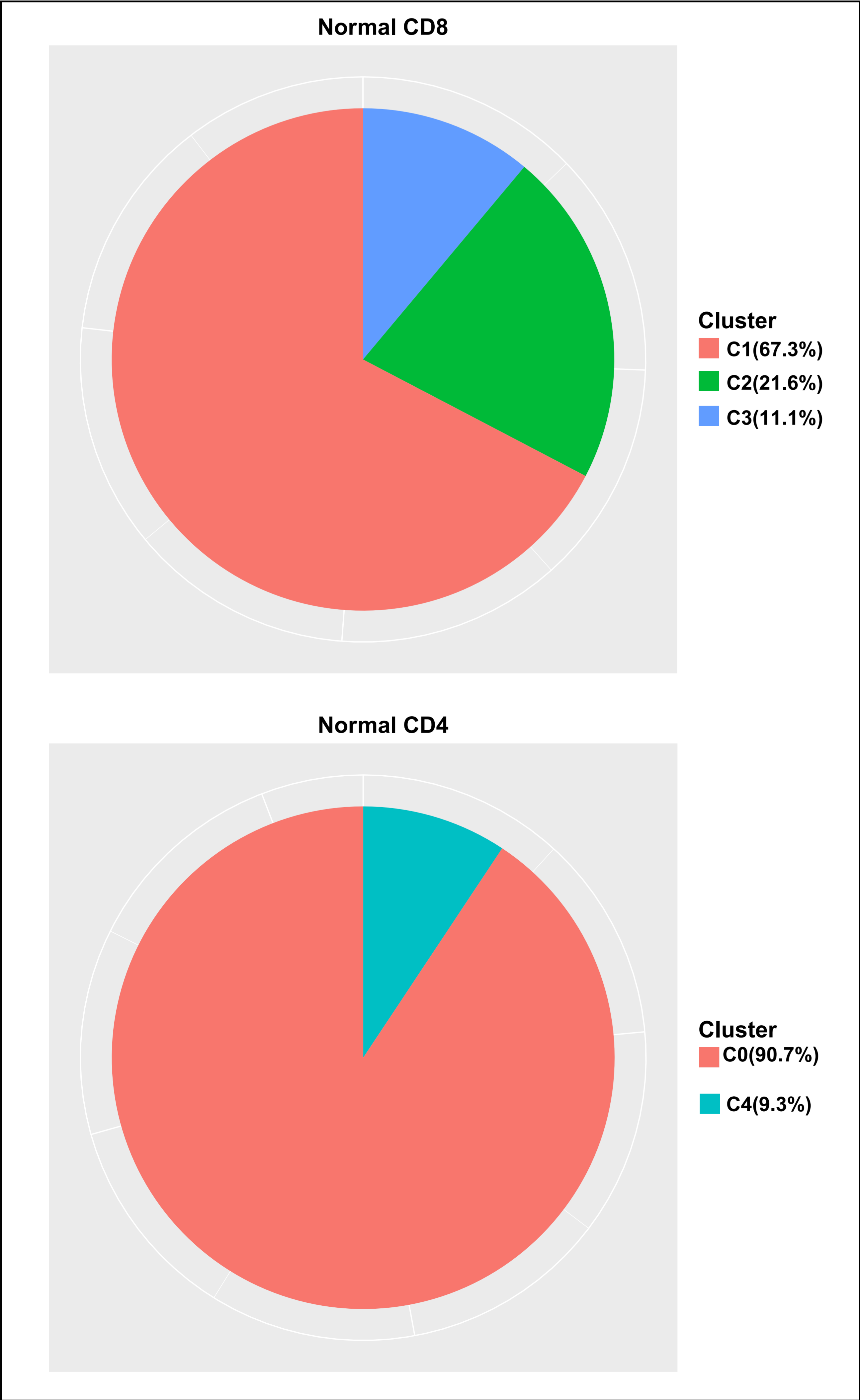

D

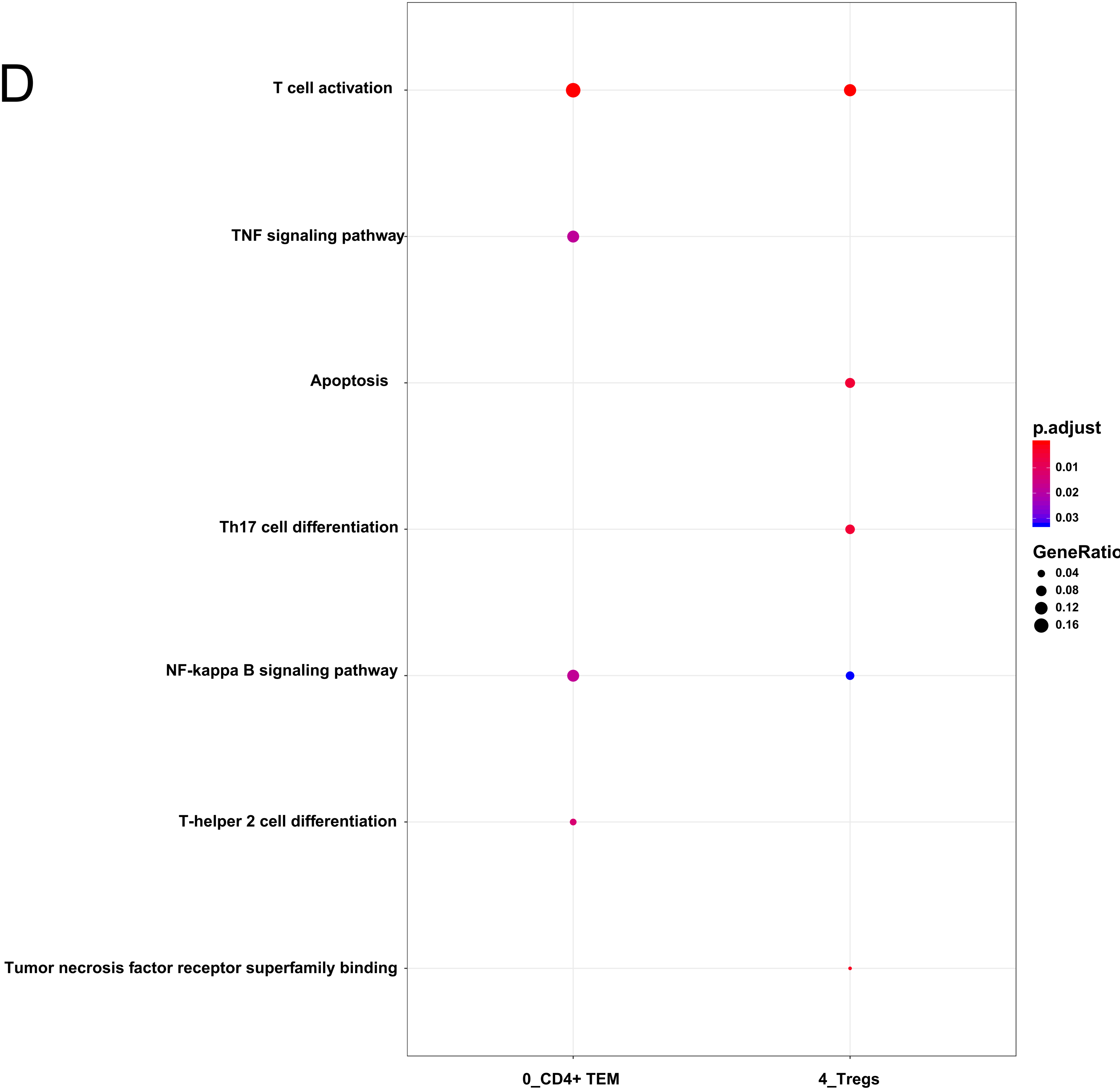

Supplement: Supplementary file 6 — Fig S6. Supplemental analysis for normal tissues. [file MOL2-15-866-s010.pdf]

A

| Gene    | R        | P value |
|---------|----------|---------|
| CXCL13  | 0.261706 | 0       |
| GAPDH   | 0.226496 | 0       |
| CHN1    | 0.223833 | 0       |
| PDCD1   | 0.211736 | 0       |
| RBPJ    | 0.202733 | 0       |
| TNFRSF9 | 0.195307 | 0       |
| RGS1    | 0.193451 | 0       |
| CTLA4   | 0.188283 | 0       |
| TIGIT   | 0.186564 | 0       |

B

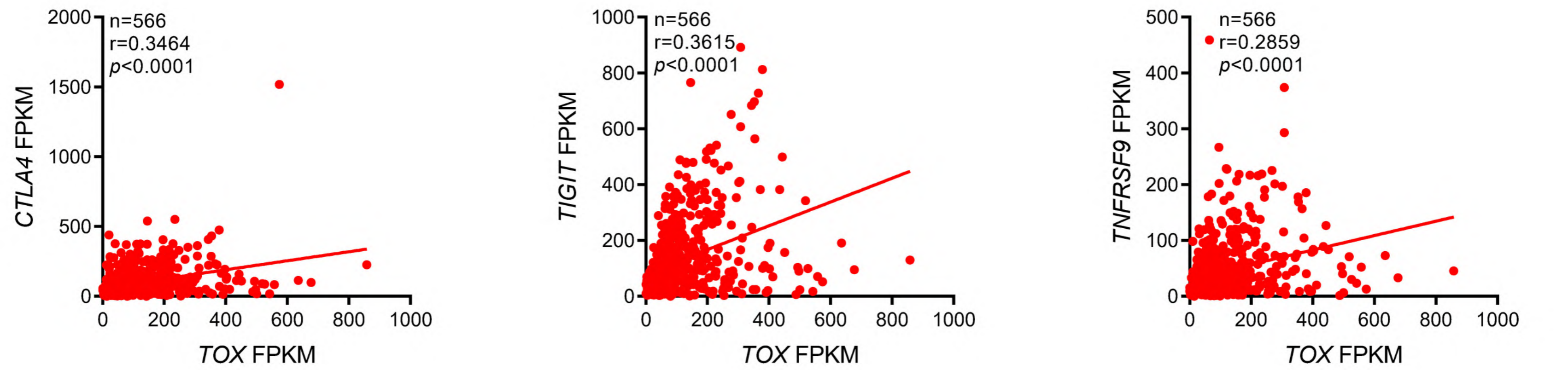

C

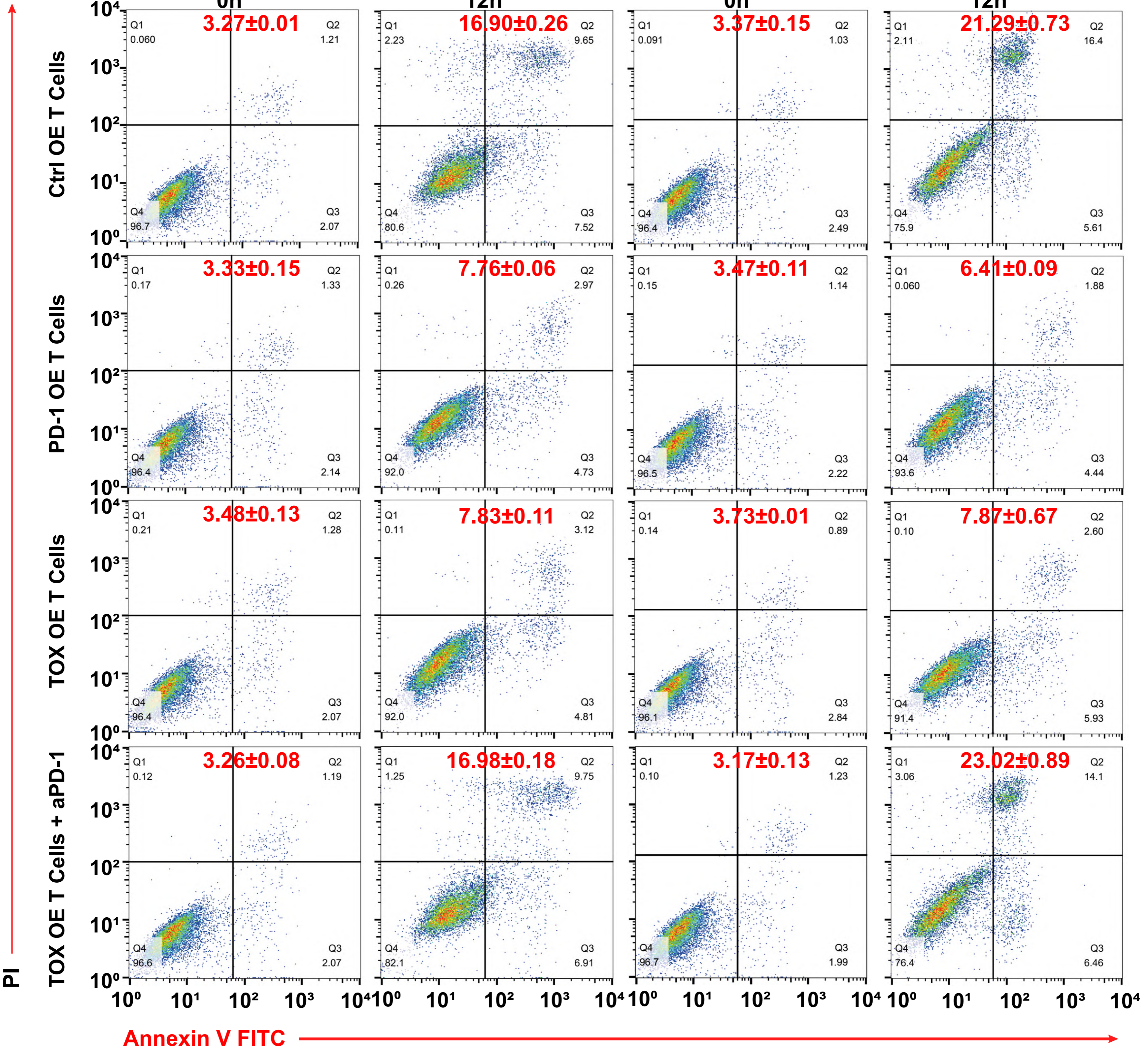

Supplement: Supplementary file 7 — Fig S7. Genes related to TOX expression and the effect of TOX on apoptosis. [file MOL2-15-866-s012.pdf]

# Regular specificity score (RSS)

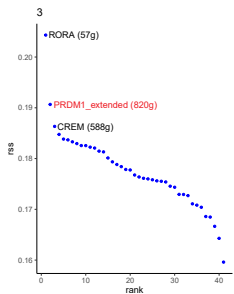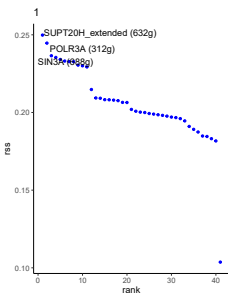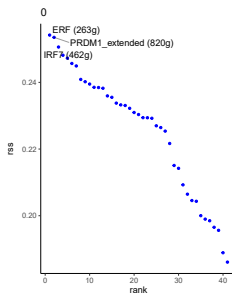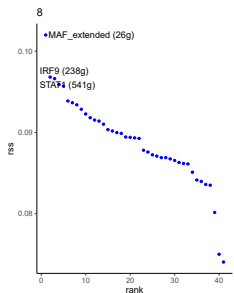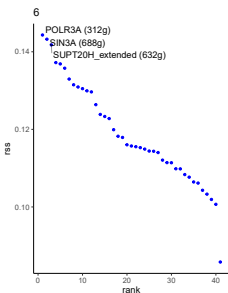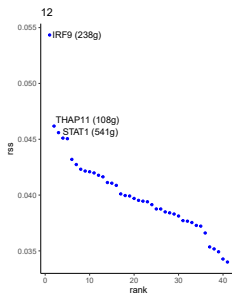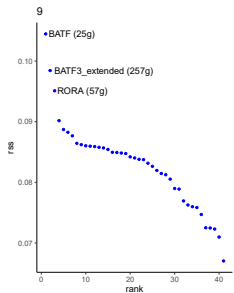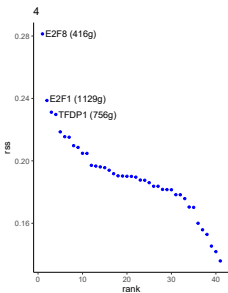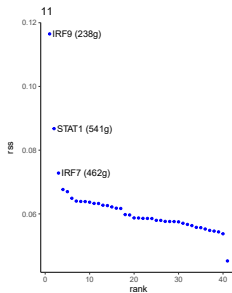

Supplement: Supplementary file 8 — Fig S8. Regular specificity scores for relevant genes driving TOX expression. [file MOL2-15-866-s013.pdf]

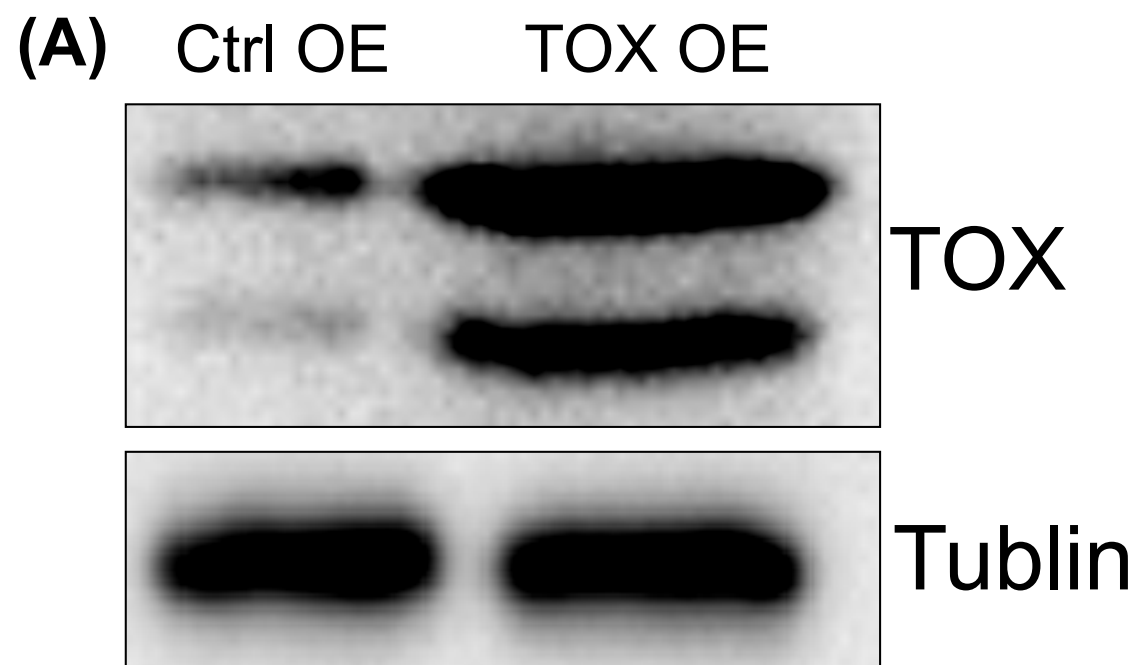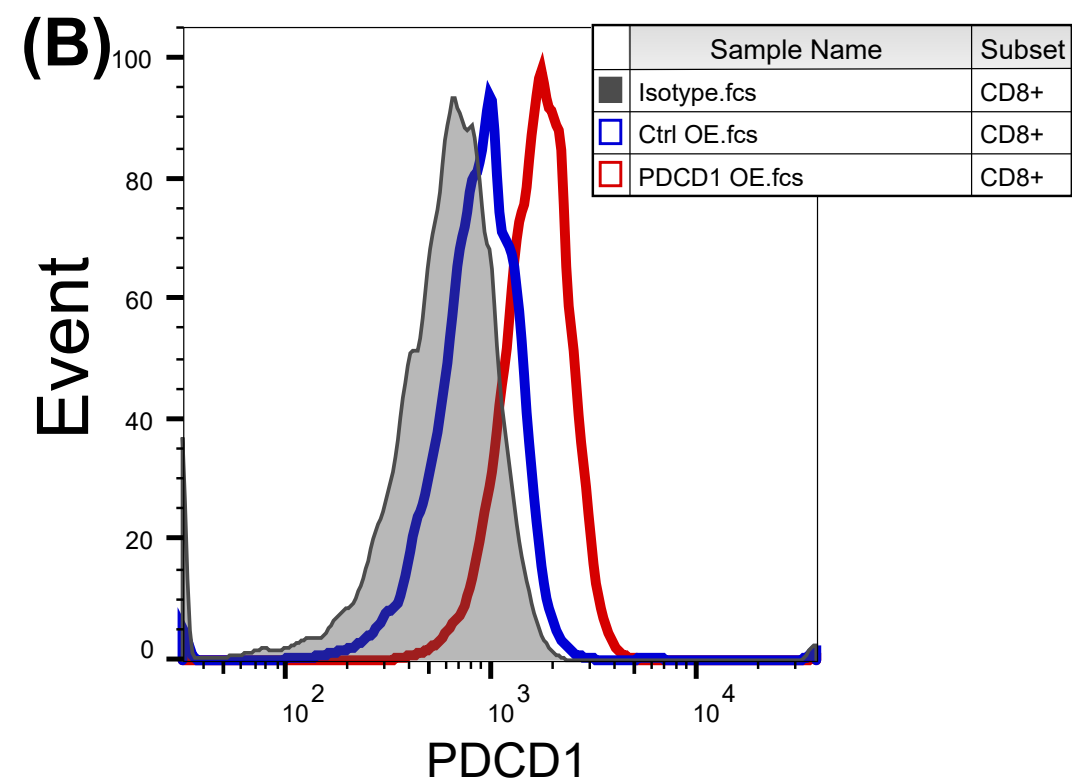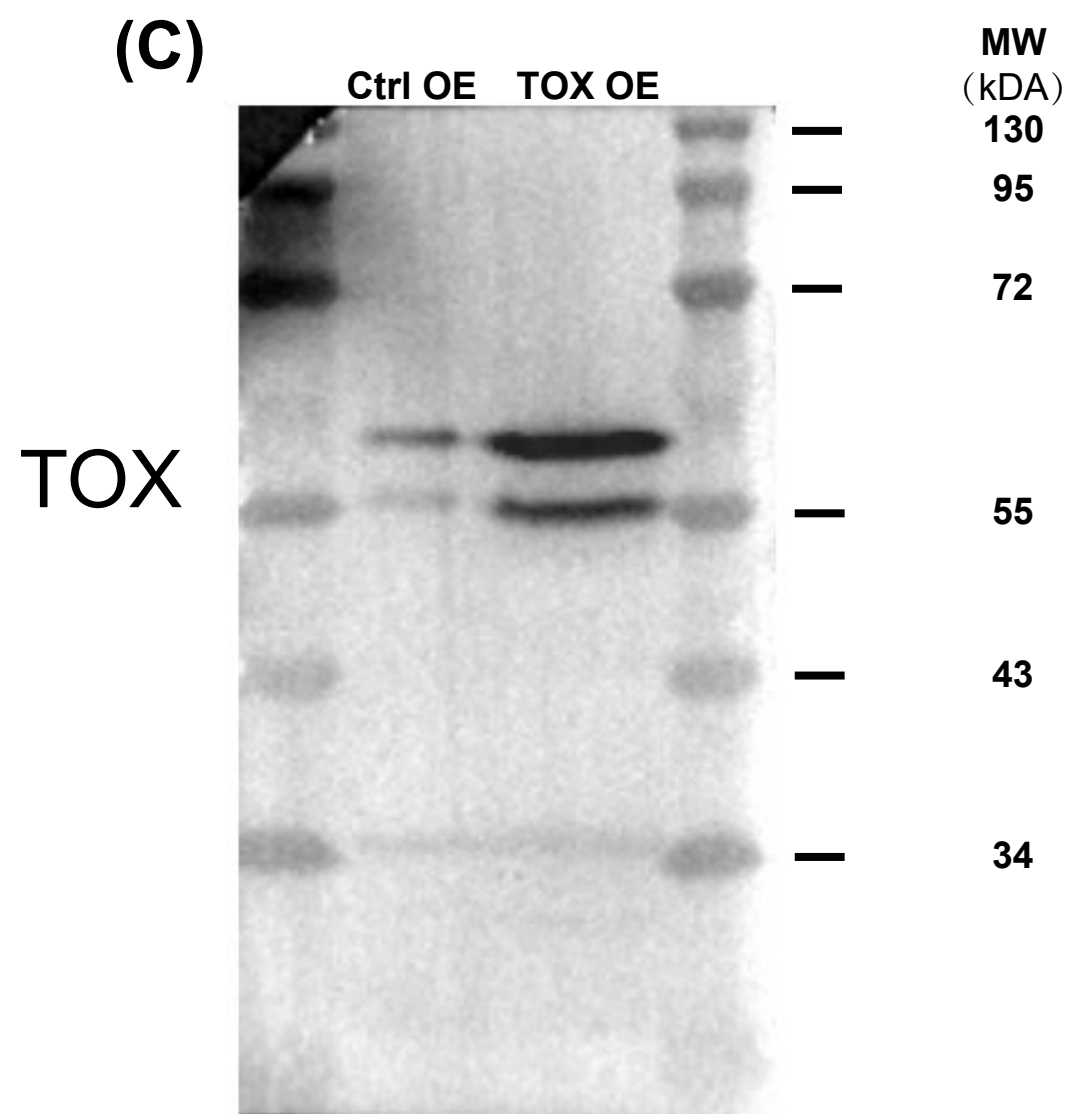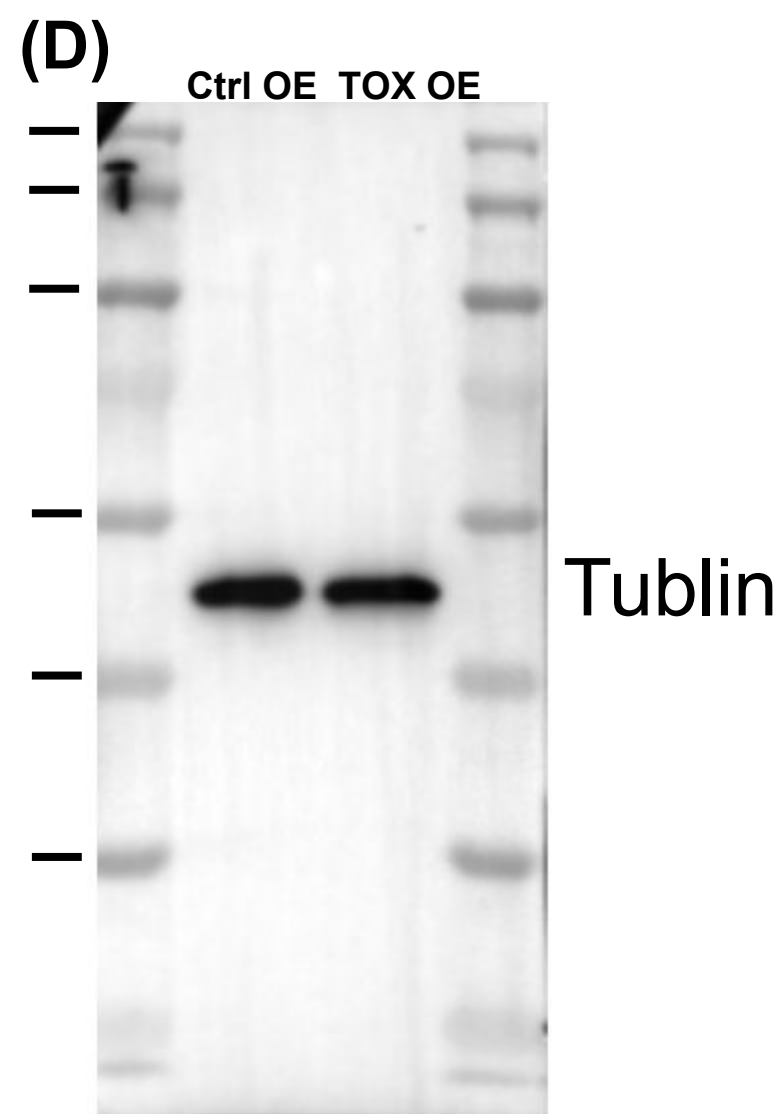

Supplement: Supplementary file 9 — Fig S9. Overexpression of marker genes. [file MOL2-15-866-s008.pdf]

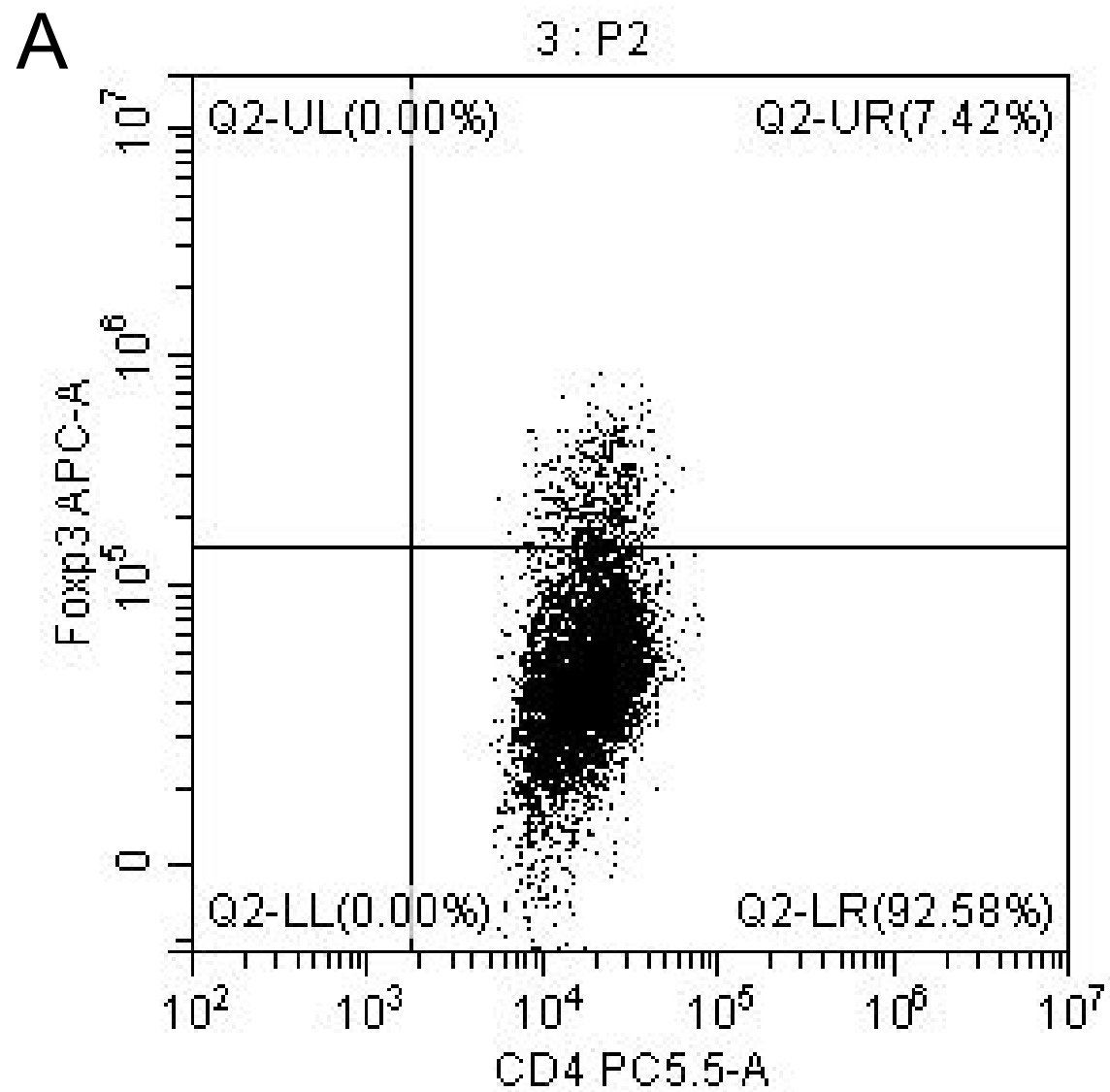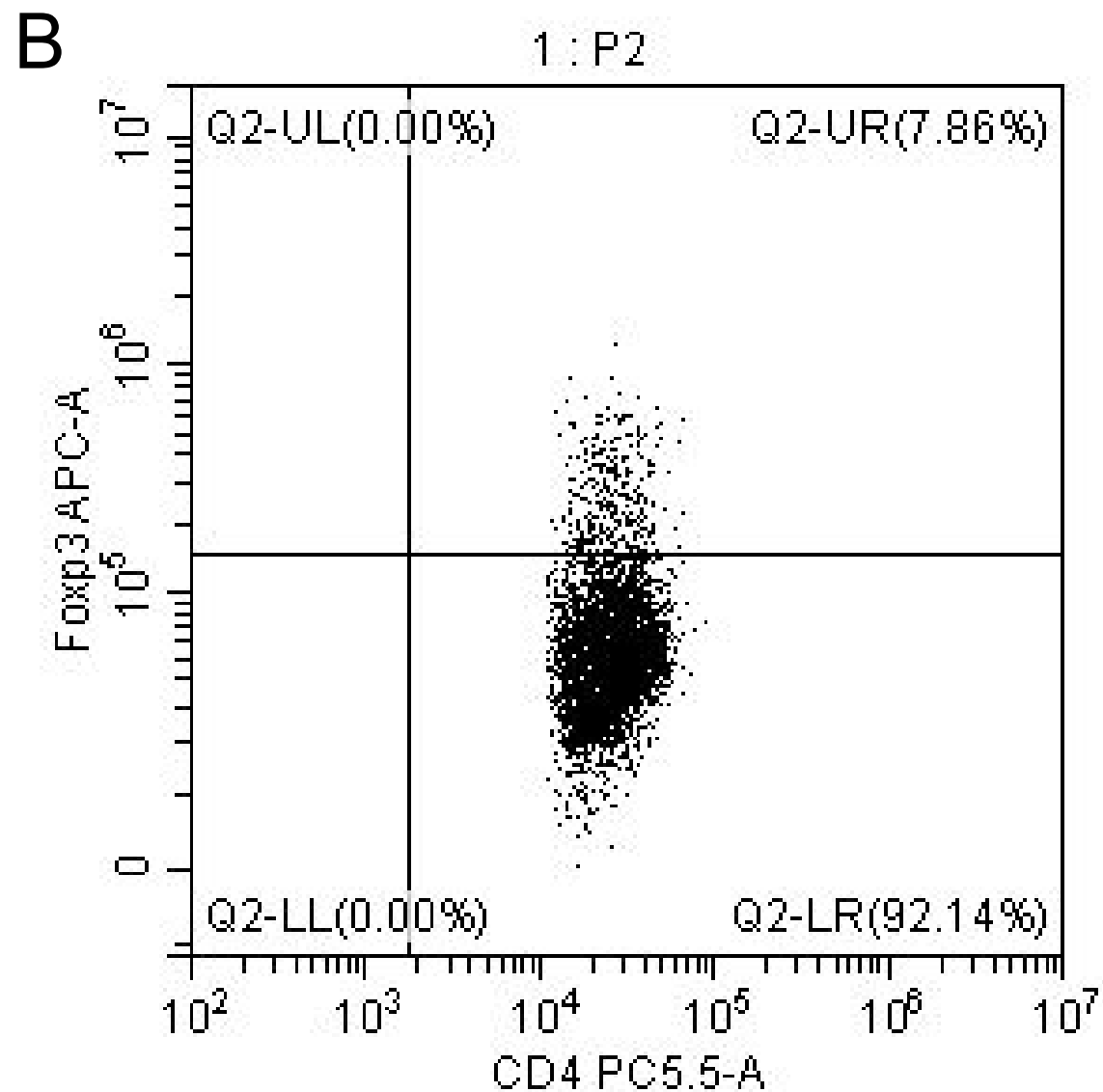

Supplement: Supplementary file 10 — Fig S10. Quantification of Foxp3+ T cells (A) Representative flow cytometry plots showing the percentage of Foxp3+ T cells in control‐overexpressing human CD4+ T cells. Human CD4+ T cells were stimulated in vitro with Human T‐Activator CD3/CD28 Dynabeads for 36h and then infected with control‐overexprssing lenti‐virus. Tregs were induced by culturing the infected T cells in the presence of recombinant human TGF‐β plus IL‐2 for 4 days. (B) Representative flow cytometry plots showing the percentage of Foxp3+ T cells in TOX‐overexpressing human CD4+ T cells. [file MOL2-15-866-s001.pdf]
